# Causes of death among people who used illicit opioids in England, 2001–18: a matched cohort study

**DOI:** 10.1016/S2468-2667(21)00254-1

**Published:** 2021-12-11

**Authors:** Dan Lewer, Thomas D Brothers, Naomi Van Hest, Matthew Hickman, Adam Holland, Prianka Padmanathan, Paola Zaninotto

**Affiliations:** a Department of Epidemiology and Public Health, University College London, London, UK; b Department of Medicine, Dalhousie University, Halifax, NS, Canada; c Population Health Sciences, Bristol Medical School, University of Bristol, Bristol, UK; d Department of Public Health, Environments and Society, London School of Hygiene & Tropical Medicine, London, UK

## Abstract

**Background:**

In many countries, the average age of people who use illicit opioids, such as heroin, is increasing. This has been suggested to be a reason for increasing numbers of opioid-related deaths seen in surveillance data. We aimed to describe causes of death among people who use illicit opioids in England, how causes of death have changed over time, and how they change with age.

**Methods:**

In this matched cohort study, we studied patients in the Clinical Practice Research Datalink with recorded illicit opioid use (defined as aged 18–64 years, with prescriptions or clinical observations that indicate use of illicit opioids) in England between Jan 1, 2001, and Oct 30, 2018. We also included a comparison group, matched (1:3) for age, sex, and general practice with no records of illicit opioid use before cohort entry. Dates and causes of death were obtained from the UK Office for National Statistics. The cohort exit date was the earliest of date of death or Oct 30, 2018. We described rates of death and calculated cause-specific standardised mortality ratios. We used Poisson regression to estimate associations between age, calendar year, and cause-specific death.

**Findings:**

We collected data for 106 789 participants with a history of illicit opioid use, with a median follow-up of 8·7 years (IQR 4·3–13·5), and 320 367 matched controls with a median follow-up of 9·5 years (5·0–14·4). 13 209 (12·4%) of 106 789 participants in the exposed cohort had died, with a standardised mortality ratio of 7·72 (95% CI 7·47–7·97). The most common causes of death were drug poisoning (4375 [33·1%] of 13 209), liver disease (1272 [9·6%]), chronic obstructive pulmonary disease (COPD; 681 [5·2%]), and suicide (645 [4·9%]). Participants with a history of illicit opioid use had higher mortality rates than the comparison group for all causes of death analysed, with highest standardised mortality ratios being seen for viral hepatitis (103·5 [95% CI 61·7–242·6]), HIV (16·7 [9·5–34·9]), and COPD (14·8 [12·6–17·6]). In the exposed cohort, at age 20 years, the rate of fatal drug poisonings was 271 (95% CI 230–313) per 100 000 person-years, accounting for 59·9% of deaths at this age, whereas the mortality rate due to non-communicable diseases was 31 (16–45) per 100 000 person-years, accounting for 6·8% of deaths at this age. Deaths due to non-communicable diseases increased more rapidly with age (1155 [95% CI 880–1431] deaths per 100 000 person-years at age 50 years; accounting for 52·0% of deaths at this age) than did deaths due to drug poisoning (507 (95% CI 452–562) per 100 000 person-years at age 50 years; accounting for 22·8% of deaths at this age). Mirroring national surveillance data, the rate of fatal drug poisonings in the exposed cohort increased from 345 (95% CI 299–391) deaths per 100 000 person-years in 2010–12 to 534 (468–600) per 100 000 person-years in 2016–18; an increase of 55%, a trend that was not explained by ageing of participants.

**Interpretation:**

People who use illicit opioids have excess risk of death across all major causes of death we analysed. Our findings suggest that population ageing is unlikely to explain the increasing number of fatal drug poisonings seen in surveillance data, but is associated with many more deaths due to non-communicable diseases.

**Funding:**

National Institute for Health Research.

## Introduction

People who use illicit opioids (eg, heroin) have an extremely high risk of death. International systematic reviews have found mortality rates in this population to be 10–15 times higher than those of the general population;^1^, ^2^, ^3^ however, the most common causes of death have changed over time. In the UK, there were so-called epidemics of heroin use in the 1980s and 1990s and people using heroin were mostly aged 18–34 years and had recently started using drugs.^4^ Most deaths in this era were due to drug overdoses, infections, and suicides.^5^, ^6^, ^7^ Although the number of new users has tailed off since the late 1990s, many people who started using opioids in the 1980s and 1990s have continued to use these drugs. In 2019, approximately 70% of people accessing treatment in England for “problematic heroin use” first used heroin before 2000, and the average age of people accessing treatment was 42 years.^8^ As the population has aged, their health needs and causes of death have changed. Cohort studies continue to show high mortality rates, but many more deaths are now caused by non-communicable diseases.^9^, ^10^

**Research in context**

**Evidence before this study**

Systematic reviews have shown that the rate of all-cause death among people who use illicit opioids is many times higher than in the general population, although this excess risk differs between countries and over time. In studies of people who used drugs in the 1980s and 1990s, sample populations are often relatively young and large proportions of deaths were attributed to drug poisoning, suicide, homicide, HIV/AIDS, and other infections. In more recent studies in settings with good availability of antiretroviral therapy, sample populations are typically older and a smaller proportion of deaths are due to infections. Surveillance data show increasing numbers of opioid-related deaths since 2010 in some regions, including the UK, Australia, and North America. One posited explanation is that the population using illicit opioids is ageing and is therefore more susceptible to opioid overdose. However, there are fewer studies investigating changes in mortality in this population over time or over the life course. We searched PubMed for studies published between Jan 1, 2001, and June 15, 2021, reporting changes in mortality rates over time or age-specific mortality rates for people who use illicit opioids. We found that the estimated association between age and risk of fatal drug poisoning varied, with some studies showing no association and others showing that risk increases with age. 13 studies showed that non-communicable diseases cause a minority of deaths in younger samples of people who use opioids, and a majority in older samples. One study in Scotland found age-specific rates of drug-related death increased between 2009 and 2018, concluding that the increase in drug-related deaths cannot be fully explained by population ageing. Other studies have not attempted to isolate changes in mortality over time from ageing in study cohorts.

**Added value of this study**

We explored causes of death in a cohort of people with a history of opioid use in England between 2001 and 2018. Our study adds to existing evidence that illicit opioid use is associated with high rates of all major causes of death, reflecting a deprived and marginalised population with multiple determinants of poor health throughout life. It also adds to existing evidence that the proportion of deaths related to non-communicable diseases increases with age, but that recent increases in the rate of deaths due to drug poisoning are unlikely to be explained by an ageing cohort.

**Implications of all the available evidence**

Increases in opioid-related deaths in the UK over the past decade are unlikely to be explained by the increasing average age of people who use drugs. However, population ageing is likely to mean that people using opioids have more complex health needs and an increased risk of death due to non-communicable diseases. Services that support this population, including community drug treatment services, will need resources to provide appropriate care for clients with increasing and often unmet health needs.

Alongside ageing, another feature of the health of this population is an increase in the number of fatal drug poisonings over the past 10 years. The number of deaths in England for which an opiate has been mentioned on the death certificate has increased by 54% between 2010 and 2020, from 1384 to 2138 deaths per year.^11^ Opioid-related deaths are also increasing in other countries, including Scotland, Australia, the USA, and Canada. The causes of this crisis differ between countries. For example, in the USA and Canada the increase in opioid-related deaths seen over the past 10 years is linked to synthetic opioids including fentanyls^12^, ^13^ in the illicit drug market, but these drugs are not yet common in the UK. One explanation proposed for increasing numbers of opioid-related deaths in the UK is that the long-term trend of ageing and increasing frailty in this population is leading to increased risk of death after using respiratory depressants such as opioids.^14^ However, most surveillance data are from national death records, and determining whether the increasing deaths are due to population ageing, more people using drugs, or environmental factors, is difficult.

Drug treatment services in the UK have experienced disinvestment and a 2020 government-sponsored review found they are struggling to meet their clients' basic needs.^15^ A review of the causes of death in this population and changes over time could inform a programme of reinvestment.

We used data from a cohort of people who use illicit opioids in England to address the following questions: what is the mortality rate among people who use illicit opioids in England? How does it compare with that among people who do not use these drugs? What are the most common causes of death in this opioid-using population? How have all-cause and cause-specific mortality rates changed in the past 20 years, and are changes explained by population ageing?

## Methods

### Study design

We did a matched cohort study of the rate of all-cause and cause-specific death, comparing people with a history of using illicit opioids with a group of individuals with no recorded history of using illicit opioids matched for age, sex, and general practice in England. We used patient-level data from general practices in England to model mortality rates in these two populations.

The study was approved by the Medicines and Healthcare products Regulatory Agency (UK) Independent Scientific Advisory Committee (number 19_142R, under Section 251; NHS Social Care Act 2006). This study is based in part on data from the Clinical Practice Research Datalink (CPRD), obtained under licence from the UK Medicines and Healthcare products Regulatory Agency. The data were provided by patients and collected by the UK National Health Service (NHS) as part of their care and support. Individual patient consent is not required for this analysis.

### Data source

We used data from the CPRD Gold and Aurum.^16^, ^17^ These research databases contain pseudonymised patient-level data from general practice clinical systems in England, with the CRPD Gold drawing data from general practices that use one software package and Aurum drawing data from practices that use a different software package. CRPD Gold covers 8% of the population of England and CRPD Aurum covers 13% of the population. These databases are representative of the population of England in terms of age, sex, ethnicity, and deprivation status. Patients in some practices are not linked to the national mortality database and we did not include these individuals. A flow chart showing the derivation of our sample is in the appendix (p 7).

Participants with a history of using illicit opioids were defined as those aged 18–64 years at study entry with a prescription of opioid agonist therapy (methadone or buprenorphine) or clinical observations such as heroin dependence. We have previously published a full codelist and validation showing that this sample has similar demographic characteristics and all-cause mortality rates as other samples of people who use illicit opioids (eg, heroin) or are receiving opioid agonist therapy.^18^ The entry date was Jan 1, 2001; 12 months after entry to CPRD; or the first record of illicit opioid use. We used a washout of 12 months to avoid the unusual period after joining a database such as CPRD because this period often coincides with registration at a doctor's surgery and might be associated with poor health, diagnosis, or recording of pre-existing health problems.^19^

For each participant, we sampled with replacement three individuals of the same sex and age (within 3 years) and from the same general practice, with no previous records of illicit opioid use in CPRD. The matched participants were assigned the same cohort entry date as the corresponding individual with a history of opioid use. This process is called exposure density sampling,^20^ and is designed to minimise biases related to the date of cohort entry.

All participants were linked to death records from the UK Office for National Statistics, including the date of death and underlying cause of death. Linkage was done by NHS Digital on the basis of NHS number, sex, date of birth, and postcode.^21^

The cohort exit date was the earliest of death or Oct 30, 2018. We had death data until May 1, 2019, but decided to end the study 6 months early (on Oct 30, 2018) to minimise biases related to delayed death registration.^22^ Registration is often delayed for drug-related deaths and suicides, which are common among people who use illicit drugs and might not have been recorded during the final 6 month period of the study.

For descriptive purposes, we extracted participants' Index of Multiple Deprivation^23^ (a composite measure of income, employment, education, health, crime, local services, and environment in each participant's neighbourhood), last-recorded smoking status before cohort entry, and body-mass index at entry to the CRPD.

### Cause of death grouping

We classified deaths using the International Classification of Diseases 10th edition (ICD-10) code for the underlying cause of death. We first identified deaths due to drug poisoning using the Office for National Statistics definition.^11^ We grouped the remaining deaths by diseases that were either identified as major causes of premature mortality in the general population of England in a previous study,^24^ or identified as major causes of death in a previous study of people in treatment for heroin dependency in south London, UK.^10^ The groups were as follows: infections (with the subgroups of HIV and viral hepatitis), cancers (with the subgroups of digestive, respiratory, lymphoid and haematopoietic, female genital, and breast), diseases of the nervous system, circulatory diseases (with the subgroups of ischaemic heart disease and cerebrovascular disease), respiratory diseases (with the subgroups of chronic obstructive pulmonary disease [COPD] and influenza or pneumonia), digestive diseases (with the subgroup of liver), and external causes (with the subgroups of accidents and suicide, excluding drug poisoning). We also created a higher level and mutually exclusive classification for use in analysis of time and age trends, which comprised drug poisoning, circulatory diseases, COPD, other respiratory diseases, respiratory cancers, other cancers, liver disease, and external causes. ICD-10 codes for these classifications are provided in the appendix (p 5).

### Statistical analysis

We described the characteristics of participants at baseline, stratified by history of illicit opioid use and we compared the distribution of non-matched variables using the χ^2^ test.

We expanded the follow-up period for each participant such that a new observation period began with each calendar period (2001–03, 2004–06, 2007–09, 2010–12, 2013–15, and 2016–18), birthday, and every third anniversary after cohort entry. This method is called Lexis expansion^25^ and allowed us to analyse disease risk on multiple timescales. An example of data expanded in this way is given in the appendix (p 6) together with analysis code.

To calculate cause-specific standardised mortality ratios (SMRs), we calculated age-sex-calendar time period-specific mortality rates in the comparison group, and then applied these rates to the number of person-years in the exposed group. This gave the number of expected deaths. The SMR is the observed number of deaths divided by the expected number of deaths. We estimated 95% CIs using a non-parametric bootstrap method^26^ in which we resampled exposed participants with matched comparators 1000 times with replacement, calculated the SMR for each resample, and reported the 2·5% and 97·5% quantiles. We used this method because the approach most commonly used, which estimates the SE of the SMR assuming a Poisson distribution in the observed deaths, assumes no error in expected deaths, but our data have less precision, particularly for rare causes of death such as HIV.

We then estimated the independent association between calendar period and mortality using a Poisson model for each cause of death. The dependent variable was the count of deaths, and the independent variables were opioid history (ie, history of using illicit opioids or not), age (linear and quadratic terms), sex, an interaction term between opioid history and calendar time period, an interaction term between opioid history and time after cohort entry, and an offset for the log follow-up time. We used the marginal rates from the model to estimate the number of deaths in a cohort of 100 000 individuals with the same characteristics as the entire sample of people who use opioids (in terms of age, sex, and time after cohort entry) in each calendar period. We used the same approach to estimate age-specific mortality rates, standardised by calendar year, time after cohort entry, and sex, and we used these estimates to compare mortality rates due to drug poisoning with non-communicable diseases at 5-year age intervals.

In a further analysis, we investigated the key policy and research question of whether population ageing is contributing to the increasing number of drug-related deaths seen in national surveillance data. We investigated this question by applying the marginal rates from our model to the age structure of people who use illicit opioids in England. This age structure was based on data from the Unlinked Anonymous Monitoring Survey of People who Inject Drugs, supplied by Public Health England (this population structure is shown graphically in the appendix [p 11]).^27^ In this population, the proportion who were younger than 40 years was 89% in 2001 and 52% in 2018. Assuming that population ageing is the only variable, this analysis shows how population ageing might have contributed to changes in cause-specific mortality rates over time.

We did all analyses using R (version 3.6.2).

### Role of the funding source

The funder of the study had no role in study design, data collection, data analysis, data interpretation, or writing of the report.

## Results

This study included data for 106 789 participants with a history of using illicit opioids, with a median of 8·7 years (IQR 4·3–13·5) of follow-up. Data for 82 241 (77·0%) of 106 789 participants were extracted from CPRD Aurum, and the remainder were extracted from CRPD Gold. 32 998 (30·9%) participants were female, 73 791 (69·1%) were male, and the median age at cohort entry was 35·1 years (IQR 29·0–42·3). We also included 320 367 matched controls with a median follow-up of 9·5 years (IQR 5·0–14·4). Participants with a history of using illicit opioids were more likely to live in deprived areas, be current smokers, be underweight, and have White ethnicity and less likely to be overweight or obese than the comparison cohort (table 1).

**Table 1:** Baseline characteristics of participants at time of entry into study

		**Individuals with a history of using illicit opioids (n=106 789)**	**Matched comparison group (n=320 367)**
Date of cohort entry			
	2001–03	25 229 (23·6%)	75 687 (23·6%)
	2004–06	15 429 (14·4%)	46 287 (14·4%)
	2007–09	17 716 (16·6%)	53 148 (16·6%)
	2010–12	17 635 (16·5%)	52 905 (16·5%)
	2013–15	16 688 (15·6%)	50 064 (15·6%)
	2016–18	14 092 (13·2%)	42 276 (13·2%)
Duration of follow-up, years		8·7 (4·3–13·5)	9·5 (5·0–14·4)
Age at entry, years		35·1 (29·0–42·3)	35·1 (29·0–42·4)
Sex			
	Female	32 998 (30·9%)	98 994 (30·9%)
	Male	73 791 (69·1%)	221 373 (69·1%)
Region of residence at entry			
	North West	22 274 (20·9%)	66 822 (20·9%)
	South West	20 187 (18·9%)	60 561 (18·9%)
	West Midlands	16 910 (15·8%)	50 730 (15·8%)
	London	14 694 (13·8%)	44 082 (13·8%)
	South Central	9699 (9·1%)	29 097 (9·1%)
	South East Coast	5668 (5·3%)	17 004 (5·3%)
	North East	5581 (5·2%)	16 743 (5·2%)
	Yorkshire and the Humber	4844 (4·5%)	14 532 (4·5%)
	East of England	4640 (4·3%)	13 920 (4·3%)
	East Midlands	2243 (2·1%)	6729 (2·1%)
	Missing	49 (<0·1%)	147 (<0·1%)
Index of Multiple Deprivation quintile of home address at entry*			
	1 (least deprived)	7412 (6·9%)	44 051 (13·8%)
	2	11 361 (10·6%)	52 047 (16·2%)
	3	16 339 (15·3%)	57 411 (17·9%)
	4	26 090 (24·4%)	73 151 (22·8%)
	5 (most deprived)	45 396 (42·5%)	93 268 (29·1%)
	Missing	191 (0·2%)	439 (0·1%)
Ethnicity*†			
	White (British, Irish, or other)	93 445 (87·5%)	224 416 (70·0%)
	Mixed or multiple ethnic groups	1416 (1·3%)	3231 (1·0%)
	Asian or Asian British	2489 (2·3%)	14 634 (4·6%)
	Black, African, Caribbean, or Black British	2378 (2·2%)	11 685 (3·6%)
	Other ethnic group	1601 (1·5%)	7873 (2·5%)
	Unknown	5460 (5·1%)	58 528 (18·3%)
Smoking status at entry*			
	Never smoker	7295 (6·8%)	146 491 (45·7%)
	Ex-smoker	7011 (6·6%)	39 681 (12·4%)
	Current smoker	83 483 (78·2%)	107 835 (33·7%)
	Missing	9000 (8·4%)	26 360 (8·2%)
Body-mass index at entry, kg/m^2^*			
	Underweight (<18·5)	5468 (5·1%)	6893 (2·2%)
	Healthy (18·5–24·9)	44 480 (41·7%)	106 196 (33·1%)
	Overweight (25·0–29·9)	20 300 (19·0%)	83 523 (26·1%)
	Obese (30·0–39·9)	11 087 (10·4%)	48 468 (15·1%)
	Severely obese (≥40)	1762 (1·6%)	7173 (2·2%)
	Missing	23 692 (22·2%)	68 114 (21·3%)
Died during follow-up		13 209 (12·4%)	5914 (1·8%)

Data are n (%) or median (IQR).

* The exposed (opioid) and unexposed (comparison) groups have different distributions of these variables, with p<0·0001 using a χ^2^ test.

† Ethnicity is derived from primary care, hospital admission, and hospital outpatient records. The most commonly recorded value has been used, or where values were tied the most recently recorded tied value was used. The opioid group has less unknown data due to higher rates of hospital admission.

**Figure 1:**
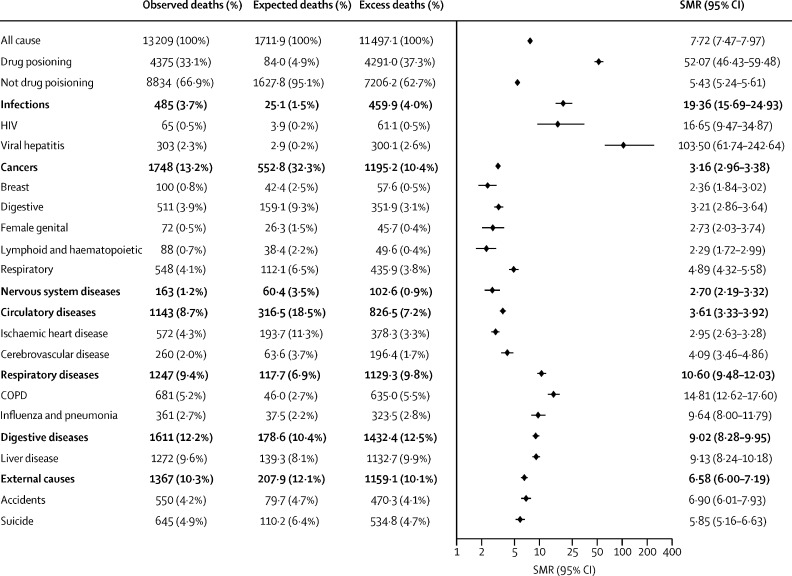
Causes of death in a cohort of 106 789 people who used illicit opioids, between 2001 and 2018, with SMRs compared with a matched comparison group with no history of illicit opioid use Median follow-up was 8·7 years. COPD=chronic obstructive pulmonary disease. SMR=standardised mortality ratio.

During the follow-up period, 13 209 (12·4%) participants with a history of using illicit opioids died. On the basis of mortality rates in the comparison group, we expected 1712 deaths in the exposed group, giving an SMR of 7·72 (95% CI 7·47–7·97). 4375 (33·1%) of 13 209 deaths were due to drug poisoning. Of the remaining deaths, the most common causes of deaths were liver disease (1272 [9·6%]), COPD (681 [5·2%]), and suicide (645 [4·9%]). The mortality rate among participants with a history of using illicit opioids was higher than in the comparison group for all causes of death we examined (figure 1). The diseases with the highest SMRs were viral hepatitis (103·5 [95% CI 61·7–242·6], HIV (16·7 [9·5–34·9]), and COPD (14·8 [12·6–17·6]). Results stratified by sex are in the appendix (pp 9–10).

**Figure 2:**
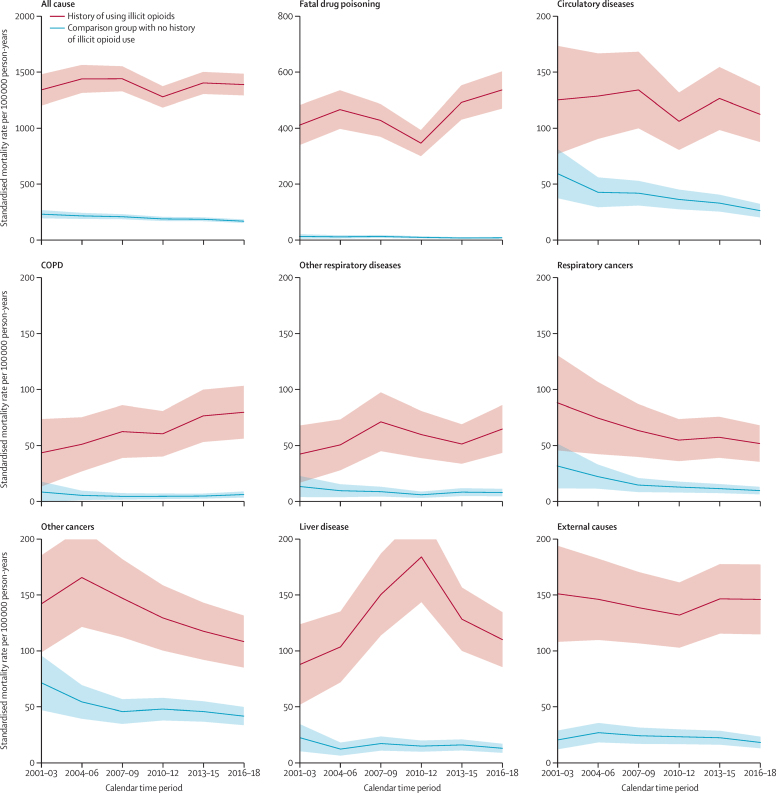
Cause-specific mortality rate by calendar time period, comparing participants with and without a history of using illicit opioids, standardised for age, duration after cohort entry, and sex Solid lines indicate point estimates and shaded areas show 95% CIs. COPD=chronic obstructive pulmonary disease.

After adjusting for age, sex, and time after cohort entry, the all-cause mortality rate among people with a history of illicit opioid use was approximately constant over time (figure 2). The all-cause mortality rate was 1335 (95% CI 1196–1474) deaths per 100 000 person-years in 2001–03 and 1383 (1287–1479) deaths per 100 000 person-years in 2016–18; whereas the all-cause mortality rate in the comparison group reduced from 225 (189–261) deaths per 100 000 person-years in 2001–03 to 162 (147–177) deaths per 100 000 person-years in 2016–18.

Among participants with a history of illicit opioid use, the mortality rate due to drug poisoning was at a minimum of 345 (299–391) deaths per 100 000 person-years in 2010–12 and increased to 534 (468–600) per 100 000 person-years in 2016–18; an increase of 55%. The mortality rate due to liver disease increased until 2010–12 and then decreased until 2016–18. The mortality rate due to COPD almost doubled between 2001–03 and 2016–18 (figure 2). Trends in mortality rates due to other diseases (circulatory diseases, other respiratory diseases, respiratory cancers, other cancers, and external causes) were either approximately constant or unclear due to wide 95% CIs.

In the comparison group, all-cause and each cause-specific mortality rate reduced between 2001–03 and 2016–18 (figure 2). In particular, the risk of deaths due to circulatory diseases reduced from 59 (37–81) deaths per 100 000 person-years in 2001–03 to 26 (20–32) per 100 000 person-years in 2016–18.

**Figure 3:**
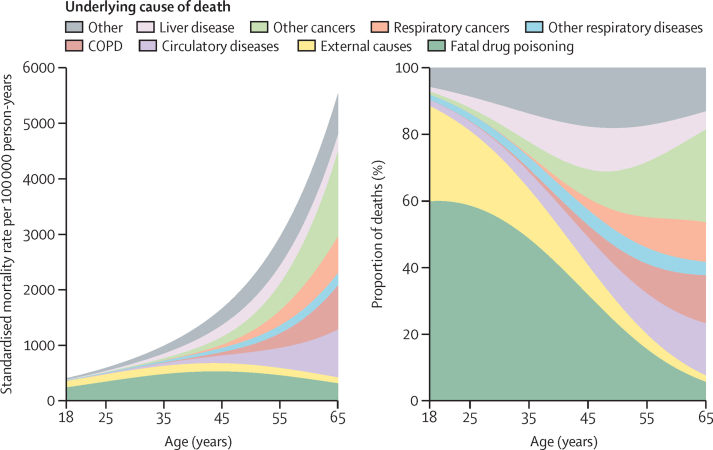
Cause-specific mortality rates by age, among participants with a history of using illicit opioids, standardised for calendar time period, duration after cohort entry, and sex COPD=chronic obstructive pulmonary disease.

We then examined age-specific mortality rates, adjusting for calendar year, time after cohort entry, and sex. Among participants with a history of illicit opioid use, deaths due to drug poisoning and external causes such as accidents and suicides were dominant at younger ages (figure 3, table 2). The rate of fatal drug poisonings increased gradually to peak at age 44 years and then decreased. Deaths due to liver disease peaked at age 58 years and then decreased. The rate of death due to circulatory diseases, respiratory diseases, and cancers all increased substantially with age, and therefore the proportion of deaths due to drug poisoning decreased. Among participants with a history of illicit opioid use, the proportion of deaths caused by drug poisoning was 59·9% at age 20 years and 22·8% at age 50 years (table 2). The rate of deaths due to non-communicable diseases (defined as cancers and circulatory, respiratory, and liver diseases) exceeded the rate of death due to drug poisoning at age 42 years.

**Table 2:** Mortality rates due to all causes, fatal drug poisoning, and non-communicable diseases at selected ages among participants with a history of using illicit opioids

	**All-cause deaths**	**Fatal drug poisonings**		Non-communicable diseases*	
	Deaths per 100 000 person-years (95% CI)	Deaths per 100 000 person-years (95% CI)	Proportion of deaths at this age	Deaths per 100 000 person-years (95% CI)	Proportion of deaths at this age
Age 20 years	452 (409–496)	271 (230–313)	59·9%	31 (16–45)	6·8%
Age 25 years	582 (538–626)	348 (306–390)	59·8%	62 (40–84)	10·7%
Age 30 years	753 (704–802)	422 (377–467)	56·0%	121 (88–155)	16·1%
Age 35 years	980 (919–1040)	483 (432–533)	49·3%	227 (172–281)	23·1%
Age 40 years	1281 (1202–1359)	520 (466–575)	40·6%	405 (311–499)	31·6%
Age 45 years	1683 (1579–1787)	529 (473–585)	31·4%	696 (533–858)	41·3%
Age 50 years	2223 (2086–2361)	507 (452–563)	22·8%	1155 (880–1431)	52·0%
Age 55 years	2953 (2766–3140)	459 (403–515)	15·5%	1860 (1408–2311)	63·0%

Mortality rates are standardised to the profile of the entire cohort of participants with a history of using illicit opioids, in terms of sex, calendar time period, and duration after cohort entry.

* Includes cancers, and circulatory, respiratory, and liver diseases.

**Figure 4:**
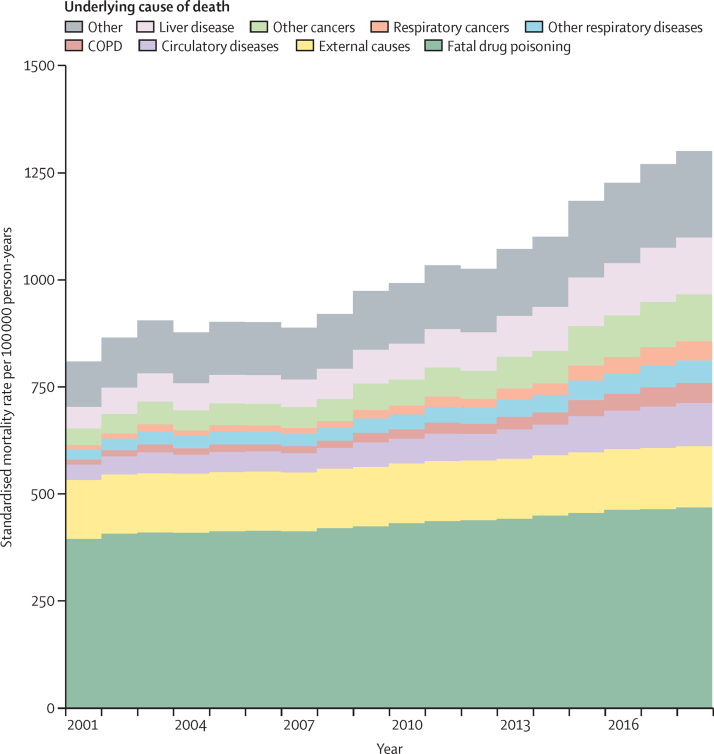
Age-specific mortality rates applied to the age structure of people who inject illicit drugs in England (from the Unlinked Anonymous Monitoring Survey of People who Inject Drugs) COPD=chronic obstructive pulmonary disease.

In a further analysis, we then applied age-specific mortality rates in our cohort to the likely age structure of the population to estimate the independent effect of population ageing on patterns of death. Comparing data from 2010 (when the rate of opioid-related deaths was lowest in the population^11^) to 2018, population ageing was associated with an increase in the rate of fatal drug poisonings from 432 (95% CI 380–483) deaths per 100 000 person-years to 468 (416–521) deaths per 100 000 person-years; an increase of 9% (figure 4; appendix p 17). Over the same period, deaths due to non-communicable diseases (ie, circulatory, respiratory, and liver diseases, and cancers combined) increased from 279 (208–351) deaths per 100 000 person-years to 487 (373–600) deaths per 100 000 person-years; an increase of 74% (figure 4; appendix p 17). Between 2001 and 2018, population ageing was associated with a tripling in the rate of death due to non-communicable diseases (figure 4; appendix p 17).

## Discussion

Among people with a history of illicit opioid use in England, we found that all major causes of death are more frequent than among people of the same age and sex in the general population; the risk of fatal drug poisoning has increased since 2010–12 and this is not explained by population ageing; and non-communicable diseases together cause more excess deaths than drug poisonings, and this difference is likely to widen with population ageing.

The increasing number of fatal drug poisonings over time, as seen in national surveillance reports in England, was also clear in our cohort. Several hypotheses have been proposed to explain this increase.^14^, ^15^, ^28^ These hypotheses include an ageing cohort of people who use drugs, increasing availability and purity of heroin, increasing poly-drug use, and reducing government investment in community drug treatment and other services that support this population. National surveillance reports usually do not include a denominator (ie, the number of people using drugs among whom the deaths occur), which means it is difficult to determine whether the trend is because of increasing risk or increasing number of people using drugs. Our results show that the rate of fatal drug poisoning is increasing, suggesting that participants in this population are at increased risk of death. The increasing rate of fatal drug poisoning between 2010–12 and 2016–18 in our cohort was not explained by ageing of participants, suggesting that the increasing number of fatal drug poisonings over the past 10 years in the population is not explained by population ageing.

The rate of death due to liver disease reduced in the exposed cohort after 2010–12, which might be because of the roll-out of direct-acting antiviral treatment for hepatitis C from 2014. Historically, hepatitis C has been common among people who use illicit opioids because of transmission when sharing injecting equipment.^27^ National surveillance data also show that the number of deaths due to hepatitis C-related liver disease decreased by 25% between 2015 and 2019, and the prevalence of hepatitis C infection among people who inject drugs is decreasing.^29^

We found increasing mortality rates due to COPD across the study period, which persisted after adjusting for ageing in the study population. Deaths due to respiratory cancers did not increase in parallel. This observation might suggest that the increase in COPD-related deaths is caused by increases in smoking crack cocaine^30^ and other drugs that damage the lungs through particles and thermal injury^31^ (ie, the mechanism does not appear to include carcinogenesis).

Non-communicable diseases are likely to become more important in this population as the average age increases. Historically, research has focused on prevention of overdoses, infections, and crime, and there are effective and cost-effective interventions that target these outcomes.^32^, ^33^, ^34^ However, little research has been done into interventions that can improve access to care for non-communicable disease, despite well documented barriers.^35^, ^36^ Community drug treatment services are sometimes the only point of contact between people who use illicit opioids and health services, and cuts in funding have meant that these services now provide a narrow range of services with little scope for holistic care.^15^ These services need resources to care for clients with increasing health and social needs.

Our study has several limitations. First, our sample of patients from general practices might not represent the population of people who use illicit opioids in England. In a validation study,^18^ we found the cohort had similar demographic characteristics and mortality rates as other studies of people who use illicit opioids, and 90% of patients being treated in hospital who received a diagnosis of opioid dependence were also captured by primary care records. This suggests that the cohort represents people with more severe opioid use or dependence but might under-represent people who use opioids for shorter periods, less frequently, or have not sought treatment. These groups might be less likely to disclose illicit drug use to their doctor and are therefore less likely to be included in this study than those with more severe use. Therefore, our results might not be generalisable to all users of illicit opioids.

Second, we did not have longitudinal data on progression of opioid dependence or cessation of drug use. We mitigated this limitation by accounting for the time after cohort entry in our model, which allows adjustment for differential changes in mortality rates between the exposed and unexposed cohorts (eg, due to cessation of drug use or other factors leading to healthier lifestyle). By including these effects in our analysis, we provide more robust evidence that observed changes over time are not biased by cessation of drug use in our cohort. There are limitations to analyses of these time-varying factors. Similarly to many analyses of concurrent age, period, and cohort associations with disease risk, we expanded follow-up into discrete windows and used the independent variance (or overlaps) of these windows. This method might be sensitive to the selection of boundaries between these windows.^37^

Third, determinants of mortality among people with a history of illicit opioid use vary between countries. For example, in the USA and Canada prescription and illicit synthetic opioids have contributed to increasing opioid-related death.^12^ In many low-income and middle-income countries, opioid agonist therapy and other harm reduction measures are less available than in high-income countries.^38^ Therefore, our results are not generalisable to people who use illicit opioids in countries other than England. The transition in the predominant causes of death from drug poisoning, infections, accidents, and suicides to non-communicable diseases has also been observed among people in opioid agonist therapy in Australia, which is also a population with increasing average age.^39^, ^40^

Fourth, despite the large sample, our study did not have sufficient power to estimate changes in mortality over time with precision, particularly in cause-specific analyses. For example, we did not have sufficient precision in our estimates to determine whether mortality rates due to circulatory diseases reduced among participants with a history of using illicit opioids, as they did in the comparison group.

Fifth, the mortality rates in our sample include cohort effects that are not present in the population using illicit opioids. In particular, the average age of the sample is likely to increase more rapidly than the average age of the population using illicit opioids. To address this, we used external data on the likely age structure of the population, from the Unlinked Anonymous Monitoring Survey of People who Inject Drugs, to model the potential effect of population ageing on mortality rates. Our cohort and this unlinked database have similar target populations, but a limitation of this modelling is that the recruitment methods differ and the survey might not represent the age structure of all people who use illicit opioids.

People who use illicit opioids in England have much higher mortality rates than the general population, and this excess mortality risk exists across all causes of death. Population ageing is likely to be associated with an increasing number of deaths due to non-communicable diseases. However, the increasing number of fatal drug poisonings in the population is unlikely to be explained by population ageing, and other hypotheses now need to be explored.

## Data sharing

This study used pseudonymised patient-level data from the CPRD. To protect patient confidentiality, we cannot publish patient-level data. Other researchers can use patient-level CPRD data in a secure environment by applying to the CPRD Independent Scientific Advisory Committee. Details of the application process and conditions of access are provided by the CPRD at https://www.cprd.com/Data-access. The codelist used to select patients for our study is publicly available at https://wellcomeopenresearch.org/articles/5-282/v2.

## Declaration of interests

MH reports honoraria for speaking at meetings from Gilead, AbbVie, and MSD. AH is co-chair of the Faculty of Public Health Drugs Special Interest Group and a member of the senior research team for the Loop; and he was previously associate director of International Doctors for Healthier Drug Policy. PP was a co-applicant on a grant awarded to the University of Bristol by Bristol and Weston Hospitals Charity focusing on suicide prevention for people presenting to hospital with self-harm and harmful substance use. All other authors declare no competing interests.
