# Supplementary material for: Causes of death among people who used illicit opioids in England, 2001–18: a matched cohort study
Source: Lancet Public Health. 2021 Dec 11;7(2):e126–35. doi: 10.1016/S2468-2667(21)00254-1 (PMC8810398; doi:10.1016/S2468-2667(21)00254-1)
Supplement: Supplementary appendix [file mmc1.pdf]

THE LANCET
Public Health

Supplementary appendix

This appendix formed part of the original submission and has been peer reviewed. We post it as supplied by the authors.

Supplement to: Lewer D, Brothers TD, Van Hest N, et al. Causes of death among people who used illicit opioids in England, 2001–18: a matched cohort study. *Lancet Public Health* 2021; published online Dec 11. https://doi.org/10.1016/S2468-2667(21)00254-1.

Supplementary Information

1. Search strategy and results for research in context panel 2
2. ICD-10 codes for causes of death 5
3. Example of 'Lexis' expanded data 6
4. Flow chart showing how the sample was derived 7
5. Results of Poisson model for all-cause death 8
6. Cause-specific mortality rates stratified by sex 9
7. Age profile of participants in the Unlinked Anonymous Monitoring Survey of People who Inject Drugs...... 11
8. Comparison between cause-specific mortality rates in the general population and from study data 12
9. Association between age and cause-specific mortality in the matched comparison group 14
10. Data used in charts in the article 15
11. STROBE checklist 18
12. References for supplementary information 21

1. Search strategy and results for research in context panel

We aimed to find studies of the association between age and/or period and cause-specific mortality in cohorts of people who use illicit opioids. We searched PubMed on 15 June 2021 for studies published after 1 January 2001 with no language restrictions containing the following terms in the title or abstract:

1. heroin OR opiate* OR opioid* OR methadone OR buprenorphine OR substance abuse* OR substance misuse* OR injects drug* OR inject drug* OR injected drug* OR drug use OR drug user* OR drug-related
2. mortality OR death* OR survival
3. cause* OR disease* OR diagnosis OR ICD
4. age OR ageing
5. year OR time OR period
6. 1 AND 2 AND 3 AND (4 OR 5)

The search returned 436 publications, of which 20 included relevant results (Table 1). The panel also includes the statements: Systematic reviews have shown that the rate of all-cause death among people who use illicit opioids is many times higher than in the general population, which is supported by Larney et al.[1]; and "In studies of people who used drugs in the 1980s and 1990s, sample populations are often relatively young and large proportions of deaths were attributed to drug poisoning, suicide, homicide, HIV/AIDS, and other infections" - for example, among people in methadone treatment in Barcelona in the 1990s, 38% of deaths were due to HIV/AIDS.[2]

Table 1: Studies of the association between age and/or period and cause-specific mortality in cohort of people who use illicit opioids

First author and publication year	Title	Population and follow-up for mortality	Age	Period	Summary
McDonald 2021[3]	Increasing drug-related mortality rates over the last decade in Scotland are not just due to an ageing cohort: A retrospective longitudinal cohort study	35,065 people in Scotland with injection-related HCV diagnosed 1991-2018; follow-up 2009-2018	✓	✓	No clear association between age and rate of drug-related death. Age-specific rate of drug-related death increased over time (table 1; figure 2).
Pearce 2020[4]	Opioid agonist treatment and risk of mortality during opioid overdose public health emergency: population based retrospective cohort study	55,347 people receiving OAT in British Columbia, Canada, 1996-2018; follow-up 1996-2018	✓		The rate of drug-related death is approximately constant with age while the rate of death due to other causes increases. Proportion of deaths that were drug-related was 57% at age 20-24 and 14% at >54 (table 2).
Gao 2019[5]	Non drug-related and opioid-specific causes of 3262 deaths in Scotland's methadone-prescription clients, 2009-2015	36,606 people in Scotland with methadone prescriptions 2009-2015; follow-up 2009-2015	✓		Rate of drug-related death increased with age – rate at 45+ is approx. double rate for under 25. Rate of death due to other causes was more strongly associated with age. Proportion of deaths that were drug-related was 55% for under 25's and 22% for 45+ (derived from table 1).
Molist 2018[6]	Effect of ageing and time since first heroin and cocaine use on mortality from external and natural causes in a Spanish cohort of drug users	15,305 heroin users starting treatment in Madrid & Barcelona 1997-2007; follow-up to 1997-2008	✓		The rate of overdose and injury deaths is constant with age, while the rate due to other causes increases. Proportion of deaths due to overdoses was 53% at age 15-29 and 23% at 40-59 (table 1).

First author and publication year	Title	Population and follow-up for mortality	Age	Period	Summary
Pavarin 2017[7]	Mortality trends among heroin users treated between 1975 and 2013 in Northern Italy: Results of a longitudinal study	5,899 people starting treatment for heroin use in Northern Italy, 1975-2013; follow-up 1975-2013.		✓	The rate of death due to overdose, injury, and drug poisonings decreased over time, while the rate of death due to other causes increased (table 2).
Jerkeman 2017[8]	Death from liver disease in a cohort of injecting opioid users in a Swedish city in relation to registration for opioid substitution therapy	4,494 people using a needle exchange service in Malmo, Sweden, 1987-2011; follow-up 1987-2011	✓		At age <30, the majority of deaths are "drug-induced". At age >40, the majority of deaths are due to other causes (figure 1).
Nambiar 2015[9]	Mortality and cause of death in a cohort of people who had ever injected drugs in Glasgow: 1982-2012	456 PWID recruited through inpatient drug treatment services in Glasgow, Scotland, 1982-1993; follow-up 1982-2012	✓		No significant differences in cause-specific mortality by age (p219), due to limited power.
Larney 2015[10]	Mortality among older adults with opioid use disorders in the Veteran's Health Administration, 2000-2011	36,608 people with lifetime opioid use disorder in the Veteran's Health Administration database, US; follow-up 2000-2011	✓		Similar rates of drug-related death in those aged 50+ and <50. The proportion of deaths that are drug-related was 9.8% for those age 50+ and 25.7% for those age <50 (table 2).
Pierce 2015[11]	National record linkage study of mortality for a large cohort of opioid users ascertained by drug treatment or criminal justice sources in England, 2005-2009	198,247 opioid users from drug treatment & criminal justice databases in England, 2005-2009; follow-up 2005-2009	✓		Rate of drug-related death increased with age – rate at 45-64 is approx. double rate for 18-24. Rate of death due to other causes was more strongly associated with age. Proportion of deaths that were drug-related was 64% at age 18-24 and 25% at 45-64 (table 3).
Vajdic 2015[12]	The impact of blood-borne viruses on cause-specific mortality among opioid dependent people: An Australian population-based cohort study	29,571 people receiving OAT in New South Wales, Australia, 1993-2007; follow-up 1993-2007	✓		Deaths due to drug poisoning, suicides, and injury were not associated with age. Deaths due to cardiovascular disease, cancer, and liver disease were strongly associated with age (table 2).
Onyeka 2014[13]	Patterns and 14-year trends in mortality among illicit drug users in Finland: the HUUTI study	4,817 people in treatment for drug use in Helsinki, Finland, 1997-2008; follow-up 1997-2008	✓		The proportion of deaths due to accidental poisoning/overdose was 36.9% at age 15-24 and 15.4% at age 45+ (table 3).
Degenhardt 2014[14]	Causes of death in a cohort treated for opioid dependence between 1985 and 2005	43,789 people starting OAT in New South Wales, Australia, 1985-2005; follow-up 1985-2005	✓	✓	The proportion of deaths that are 'accidental opioid-related' was 59.5% at age <25 and 20.3% at age 45+. The proportion due to suicide and accidents also decrease with age while liver, cardiovascular, and cancers increase (table 3). The proportion of deaths that are drug-related reduces from 58% in 1985-89 to 43% in 2000-05 (table 1).
Kielland 2013[15]	All-cause and liver-related mortality in hepatitis C infected drug users followed for 33 years: a controlled study	523 PWID admitted to residential drug treatment in Norway, 1970-1984, all with stored blood; follow-up to 2008	✓		Proportion of deaths that are drug-related decreases with age, and among HCV RNA positive individuals, deaths due to liver disease increase with age (fig 3).
Gibson 2011[16]	The increasing mortality burden of liver disease among opioid-dependent people: cohort study	2,489 people starting methadone treatment for heroin dependence in New South Wales, Australia, 1980-1985; follow-up 1980-2008		✓	The rate of deaths due to suicide, drug poisoning, and accidents was approximately constant over time. The rate of death due to cancers and liver disease increase accounted for the majority of deaths by 2005/6 (fig 2).
Stenbacka 2010[17]	Mortality and cause of death among 1705 illicit drug users: a 37 year follow up	1,705 'illicit drug users' in Stockholm, 1967; follow-up to 2003	✓	✓	Abstract states "Accidents and suicide were the most common cause of death among the youngest subjects (15–24 years) and cardiovascular diseases and tumours among the oldest (≥55 years)" but this is unclear in results. Results state that accidents & suicides accounted for smaller proportion of deaths over time.
Beynon 2010[18]	Older and sicker: Changing mortality of drug users in treatment in the North West of England	People who died in drug treatment in North-West England, 2003-2008	✓		Proportion of deaths that were drug related was 45% at age <40 and 22% at age 40+. The average age at death increased.

First author and publication year	Title	Population and follow-up for mortality	Age	Period	Summary
Ferreros 2008[19]	The shifting pattern of cause-specific mortality in a cohort of human immunodeficiency virus-infected and non-infected injecting drug users	7,186 PWID attending AIDS information centres in Spain, 1987-1996; follow-up 1987-2004.		✓	Comparing cause-specific mortality rates before and after 1997, deaths due to drug poisoning and AIDS decreased while deaths due to cardiovascular, liver, and other diseases increased (table 2).
Maxwell 2005[20]	Deaths of clients in methadone treatment in Texas: 1994-2002	266 people who died while in methadone treatment in Texas, 1994-2002		✓	The proportion of deaths that were due to overdoses decreased from 24% in 1994 to 12% in 2002 (table 1).
Copeland 2004[21]	Changing patterns in causes of death in a cohort of injecting drug users, 1980-2001	667 PWID at a GP practice in Edinburgh, 1980-2001; follow-up to 2001		✓	The cohort is not big enough to see associations between age and cause of death. The main cause of death is HIV/AIDS, which increases substantially from around 1990.
Bargagli 2001[22]	Mortality among problem drug users in Rome: an 18-year follow-up study, 1980-97	11,432 'problem drug users' entering treatment in Rome, 1980-1995; 1997		✓	The rate of drug-related deaths and 'other causes' was roughly constant over time. HIV/AIDS increased substantially from the mid-1980s.

OAT = Opioid Agonist Therapy
HCV = Hepatitis C Virus
PWID = People Who Inject Drugs

2. ICD-10 codes for causes of death

We classified deaths using 3-digit ICD-10 codes for the underlying cause of death. We first classified deaths into those caused by drug poisoning and other deaths using the definition provided by the Office for National Statistics.[23] We classified the remaining deaths in subgroups that were either (a) identified as major causes of premature mortality in England in a previous study,[24] where 'major causes' were defined as diseases causing 50,000 or more deaths among people aged under 75 between 2001 and 2018; or (b) identified as major causes of death in a previous study of people in treatment for heroin dependent in South London.[25]

Table 2: ICD-10 codes used to classify the underlying cause of death

Cause	ICD-10 code of underlying cause of death
Drug poisoning	F11:16, F18:19, X40:44, X60:64, X85, Y10-14
I: Infections	A00:98, B00:99
HIV	B20:24
Viral hepatitis	B15:19
II: Cancers	C00:97, D00:48
Breast cancers	C50
Digestive cancers	C15:26
Female genital cancers	C51:58
Respiratory cancers	C30:39
Lymphoid and haemopoietic cancers	C81:96
VI: Nervous system	G00:98
IX: Circulatory diseases	I00:99
Ischaemic heart disease	I20:25
Stroke / cerebrovascular disease	I60:69
Other forms of heart disease	I30:52
X: Respiratory diseases	J00:98
COPD	J40:44
Influenza and pneumonia	J09:18
XI: Digestive diseases	K00:92
Liver diseases	K70:76
XX: External causes (ex. drug-related)	V00:97, W00:98, X00:39, X45:X59, X65:X84, X86-X99, Y00:Y09, Y15:98, U50
Accidents	V00:97, W00:98, X00:39, X45:X59
Self-harm	X65:84, Y15-34

COPD = Chronic Obstructive Pulmonary Disease

3. Example of 'Lexis' expanded data

Each participant in the cohort has dates of entry, exit, birth, and death. An example is provided in table S3.

Table 3: Example of entry and exit dates for two participants (data is generated randomly)

Participant ID	Cohort entry date	Cohort exit date	Date of birth	Died (on exit date)
379	19 November 2010	23 September 2017	3 January 1972	1
21907	16 November 2011	30 October 2018	16 November 1992	0

This data is expanded to show the duration of follow-up by calendar year, age group, and duration after cohort entry. For example, expanded data for the two participants in the table above are shown in the table below, with age groups rather than single-year-of-age for brevity.

Table 4: Example of Lexis-expanded data

Participant ID	Calendar year	Age group	Years after cohort entry	Follow-up duration (days)	Died
379	2010-2012	35-39	[0-3)	410	0
379	2010-2012	40-44	[0-3)	364	0
379	2013-2015	40-44	[0-3)	321	0
379	2013-2015	40-44	[3-6)	774	0
379	2016-2018	40-44	[3-6)	322	0
379	2016-2018	40-44	[6-9)	46	0
379	2016-2018	45-49	[6-9)	264	1
21907	2010-2012	18-24	[0-3)	412	0
21907	2013-2015	18-24	[0-3)	683	0
21907	2013-2015	18-24	[3-6)	412	0
21907	2016-2018	18-24	[3-6)	684	0
21907	2016-2018	18-24	[6-9)	2	0
21907	2016-2018	25-29	[6-9)	384	0

Code that performs this expansion is provided here:
https://github.com/danlewer/hupio/blob/main/lexis/lexis_expansion.R

4. Flow chart showing how the sample was derived

Figure 1: Flow chart showing how the sample was derived

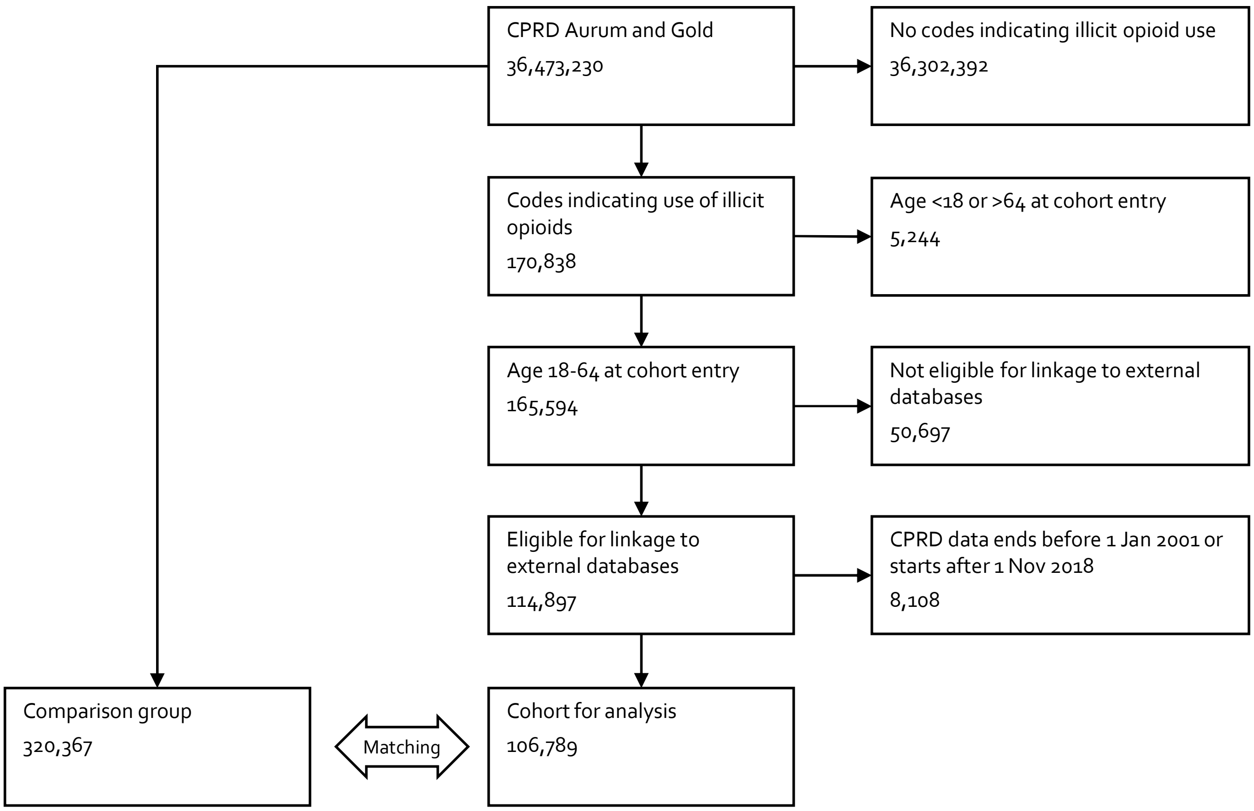

Participants not eligible for linkage to external databases are excluded at practice-level (rather than individual-level)

5. Results of Poisson model for all-cause death

Table 5: Incidence rate ratios of all-cause death

Variable	Level	Stratum*	Unadjusted	Fully adjusted
History of using illicit opioids (ref: no history)			7.216 (6.998-7.442)	8.401 (7.231-9.796)
Age (linear)			2.567 (2.516-2.619)	2.849 (2.790-2.910)
Age (quadratic)			0.778 (0.759-0.797)	1.039 (1.015-1.063)
Sex	Male (ref: female)		1.261 (1.221-1.303)	1.304 (1.262-1.347)
Calendar year	2001-03 (ref)	Comparison	1	1
	2004-06		1.096 (0.935-1.289)	0.927 (0.784-1.099)
	2007-09		1.222 (1.055-1.421)	0.892 (0.763-1.046)
	2010-12		1.321 (1.147-1.527)	0.803 (0.689-0.939)
	2013-15		1.523 (1.329-1.755)	0.787 (0.677-0.919)
	2016-18		1.647 (1.439-1.895)	0.711 (0.611-0.831)
	2001-03 (ref)	Opioids	1	1
	2004-06		1.054 (0.956-1.163)	1.069 (0.966-1.184)
	2007-09		1.136 (1.038-1.246)	1.065 (0.969-1.172)
	2010-12		1.090 (0.999-1.192)	0.942 (0.859-1.036)
	2013-15		1.305 (1.199-1.423)	1.033 (0.943-1.133)
	2016-18		1.418 (1.304-1.544)	1.022 (0.933-1.121)
Years after cohort entry	[0-3) (ref)	Comparison	1	1
	[3-6)		1.204 (1.113-1.302)	1.077 (0.992-1.171)
	[6-9)		1.373 (1.267-1.488)	1.093 (1.002-1.192)
	[9-12)		1.704 (1.568-1.851)	1.221 (1.114-1.338)
	[12-15)		1.940 (1.772-2.123)	1.225 (1.106-1.355)
	15+		2.328 (2.095-2.584)	1.322 (1.171-1.490)
	[0-3) (ref)	Opioids	1	1
	[3-6)		0.924 (0.881-0.968)	0.806 (0.768-0.847)
	[6-9)		0.964 (0.916-1.014)	0.741 (0.703-0.781)
	[9-12)		0.992 (0.938-1.049)	0.687 (0.647-0.729)
	[12-15)		1.096 (1.028-1.168)	0.643 (0.599-0.688)
	15+		1.234 (1.140-1.335)	0.620 (0.568-0.676)

* In unadjusted results, coefficients are estimated by fitting models on data stratified by exposure. In adjusted results, coefficients are estimated from models with interaction terms.

6. Cause-specific mortality rates stratified by sex

Table 6: Cause-specific mortality rates stratified by sex

Underlying cause of death	Sex	Observed deaths	Crude mortality rate per 100,000 person-years (95% CI)	Expected deaths	Standardised mortality ratio (95% CI)
All cause	All	13,209	1,374.9 (1,351.6-1,398.6)	1,711.9	7.72 (7.47-7.97)
	Female	3,465	1,187.1 (1,147.9-1,227.3)	414.1	8.37 (7.86-8.90)
	Male	9,744	1,456.9 (1,428.1-1,486.1)	1,297.7	7.51 (7.24-7.78)
Drug-related	All	4,375	455.4 (442.0-469.1)	84.0	52.07 (46.43-59.48)
	Female	971	332.7 (312.1-354.3)	17.2	56.42 (44.08-76.14)
	Male	3,404	508.9 (492.0-526.3)	66.8	50.95 (44.41-58.61)
Non-drug-related	All	8,834	919.5 (900.5-938.9)	1,627.8	5.43 (5.24-5.61)
	Female	2,494	854.5 (821.3-888.7)	396.9	6.28 (5.91-6.71)
	Male	6,340	947.9 (924.7-971.5)	1,230.9	5.15 (4.96-5.34)
I: INFECTIONS	All	485	50.5 (46.1-55.2)	25.1	19.36 (15.69-24.93)
	Female	106	36.3 (29.7-43.9)	7.7	13.78 (9.39-22.19)
	Male	379	56.7 (51.1-62.7)	17.4	21.83 (17.03-29.19)
HIV	All	65	6.8 (5.2-8.6)	3.9	16.65 (9.47-34.87)
	Female	17	5.8 (3.4-9.3)	0.6	26.23 (8.53-Inf)
	Male	48	7.2 (5.3-9.5)	3.3	14.75 (8.19-36.43)
Viral hepatitis	All	303	31.5 (28.1-35.3)	2.9	103.50 (61.74-242.64)
	Female	62	21.2 (16.3-27.2)	0.0	Inf
	Male	241	36.0 (31.6-40.9)	2.9	82.32 (48.25-188.35)
II: CANCERS	All	1,748	181.9 (173.5-190.7)	552.8	3.16 (2.96-3.38)
	Female	602	206.3 (190.1-223.4)	186.7	3.22 (2.90-3.60)
	Male	1,146	171.3 (161.6-181.6)	366.1	3.13 (2.90-3.39)
Breast	All	100	10.4 (8.5-12.7)	42.4	2.36 (1.84-3.02)
	Female	100	34.3 (27.9-41.7)	41.7	2.40 (1.85-3.06)
	Male	0	0	0.8	0 (0-0)
Digestive	All	511	53.2 (48.7-58.0)	159.1	3.21 (2.86-3.64)
	Female	100	34.3 (27.9-41.7)	36.9	2.71 (2.07-3.50)
	Male	411	61.5 (55.7-67.7)	122.2	3.36 (2.94-3.86)
Female genital	All	72	7.5 (5.9-9.4)	26.3	2.73 (2.03-3.74)
	Female	72	24.7 (19.3-31.1)	26.3	2.73 (2.00-3.72)
	Male	0	-	-	-
Lymph. & heam.	All	88	9.2 (7.3-11.3)	38.4	2.29 (1.72-2.99)
	Female	17	5.8 (3.4-9.3)	9.6	1.77 (0.92-3.16)
	Male	71	10.6 (8.3-13.4)	28.8	2.46 (1.80-3.34)
Respiratory	All	548	57.0 (52.4-62.0)	112.1	4.89 (4.32-5.58)
	Female	192	65.8 (56.8-75.8)	28.1	6.84 (5.38-8.76)

	Male	356	53.2 (47.8-59.1)	84.0	4.24 (3.64-4.95)
VI: NERVOUS SYSTEM	All	163	17.0 (14.5-19.8)	60.4	2.70 (2.19-3.32)
	Female	54	18.5 (13.9-24.1)	15.2	3.56 (2.41-5.34)
	Male	109	16.3 (13.4-19.7)	45.2	2.41 (1.90-3.09)
IX: CIRCULATORY	All	1,143	119.0 (112.2-126.1)	316.5	3.61 (3.33-3.92)
	Female	315	107.9 (96.3-120.5)	58.1	5.42 (4.54-6.53)
	Male	828	123.8 (115.5-132.5)	258.4	3.20 (2.92-3.53)
Ischaemic heart	All	572	59.5 (54.8-64.6)	193.7	2.95 (2.63-3.28)
	Female	125	42.8 (35.6-51.0)	26.2	4.77 (3.62-6.21)
	Male	447	66.8 (60.8-73.3)	167.5	2.67 (2.36-3.01)
Cerebrovascular	All	260	27.1 (23.9-30.6)	63.6	4.09 (3.46-4.86)
	Female	91	31.2 (25.1-38.3)	17.5	5.19 (3.76-7.19)
	Male	169	25.3 (21.6-29.4)	46.0	3.67 (2.91-4.65)
X: RESPIRATORY	All	1,247	129.8 (122.7-137.2)	117.7	10.60 (9.48-12.03)
	Female	393	134.6 (121.7-148.6)	34.3	11.47 (9.32-14.35)
	Male	854	127.7 (119.3-136.5)	83.4	10.24 (8.99-11.79)
COPD	All	681	70.9 (65.7-76.4)	46.0	14.81 (12.62-17.60)
	Female	220	75.4 (65.7-86.0)	15.2	14.48 (10.85-19.94)
	Male	461	68.9 (62.8-75.5)	30.8	14.96 (12.44-18.61)
Influenza & pneumonia	All	361	37.6 (33.8-41.7)	37.5	9.64 (8.00-11.79)
	Female	84	28.8 (23.0-35.6)	10.6	7.95 (5.45-11.98)
	Male	277	41.4 (36.7-46.6)	26.9	10.30 (8.13-13.35)
XI: DIGESTIVE	All	1,611	167.7 (159.6-176.1)	178.6	9.02 (8.28-9.95)
	Female	437	149.7 (136.0-164.4)	36.2	12.07 (9.78-14.80)
	Male	1,174	175.5 (165.6-185.9)	142.4	8.24 (7.37-9.17)
Liver disease	All	1,272	132.4 (125.2-139.9)	139.3	9.13 (8.24-10.18)
	Female	342	117.2 (105.1-130.3)	26.6	12.86 (10.21-16.42)
	Male	930	139.0 (130.3-148.3)	112.7	8.25 (7.35-9.37)
XX: EXTERNAL CAUSES	All	1,367	142.3 (134.8-150.0)	207.9	6.58 (6.00-7.19)
	Female	274	93.9 (83.1-105.7)	23.4	11.69 (9.21-15.08)
	Male	1,093	163.4 (153.9-173.4)	184.5	5.93 (5.41-6.55)
Accidents	All	550	57.2 (52.6-62.2)	79.7	6.90 (6.01-7.93)
	Female	112	38.4 (31.6-46.2)	12.7	8.85 (6.30-12.85)
	Male	438	65.5 (59.5-71.9)	67.1	6.53 (5.57-7.68)
Suicide	All	645	67.1 (62.1-72.5)	110.2	5.85 (5.16-6.63)
	Female	126	43.2 (36.0-51.4)	7.8	16.20 (10.95-25.73)
	Male	519	77.6 (71.1-84.6)	102.5	5.07 (4.41-5.84)

7. Age profile of participants in the Unlinked Anonymous Monitoring Survey of People who Inject Drugs

We used data from the Unlinked Anonymous Monitoring Survey of People who Inject Drugs[26] (UAM) to create an example population structure. UAM is a cross-sectional survey that recruits people who inject drugs from drug treatment services, needle and syringe programmes, and other outreach services in England, Wales, and Northern Ireland. It has run annually since the early 1990s. The primary purpose of the survey is to monitor the prevalence of HIV and hepatitis C infections in this population, but the survey also collects demographic characteristics of participants. We used this population to demonstrate the likely effect of population ageing on mortality rates among people who inject drugs. The age structure is shown in the left panel of figure S1, and for context compared to people in treatment for opiate dependence in England, 2005-2018, in the right panel, using data from the National Drug Treatment Monitoring System.[27]

Figure 2: Age of participants in the Unlinked Anonymous Monitoring Survey of People who Inject Drugs (left panel) and people in treatment for opiate dependence in England (right panel)

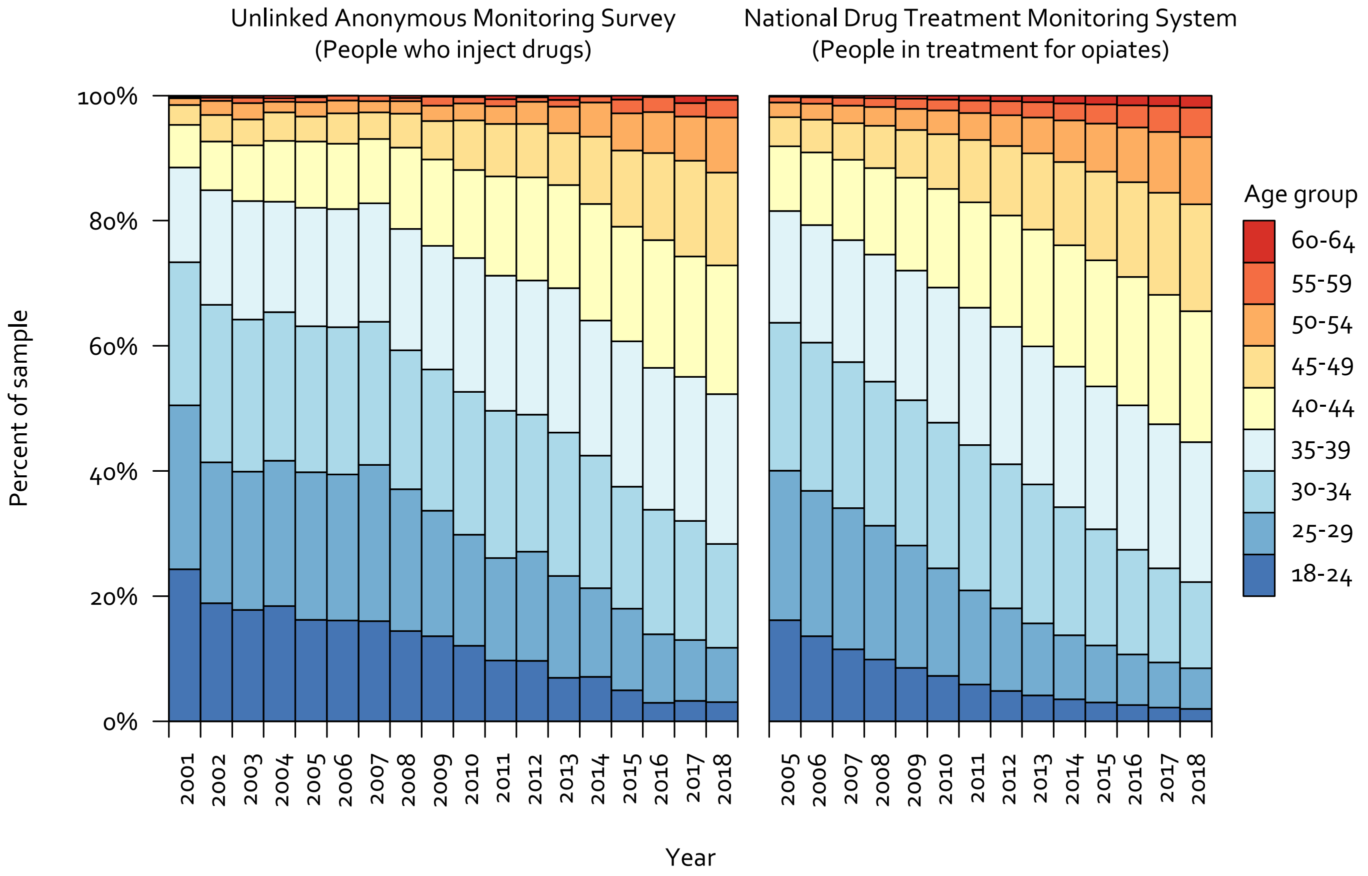

8. Comparison between cause-specific mortality rates in the general population and from study data

Figure 3: Population-rate of heroin or morphine-related death, compared to the standardized rate of death due to drug poisoning in our study

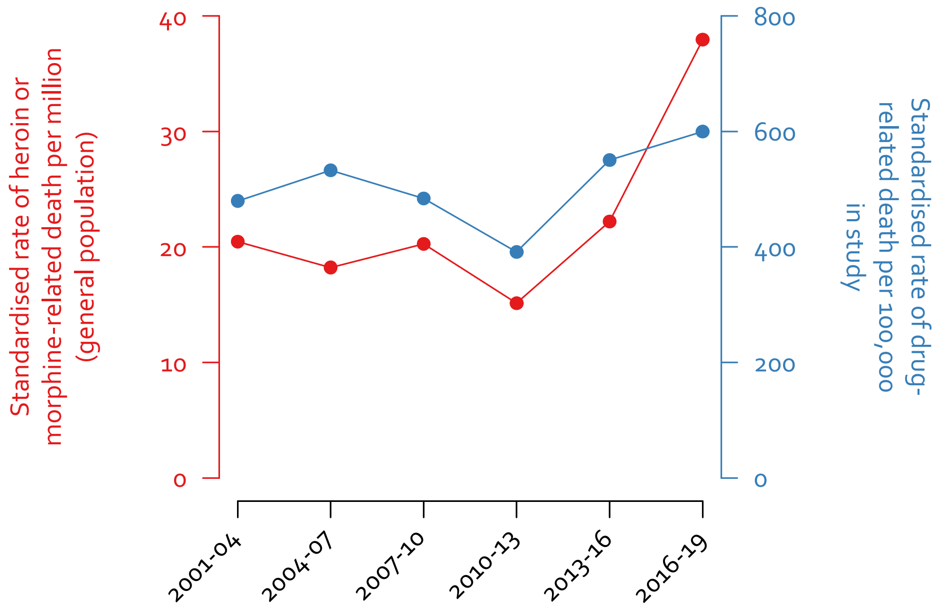

The population rate is calculated from mortality data published by ONS[23] and population estimates published by Nomisweb. Rates are directly standardised using the average population aged 15-79 between 2001 and 2019. Rates in the study are standardised using the method described in the main article. Data and code for this chart are available at https://github.com/danlewer/hupio/tree/main/general_pop_rates.

Figure 4: Cause-specific mortality rate in the general population of England and Wales, men and women, 2010-2019

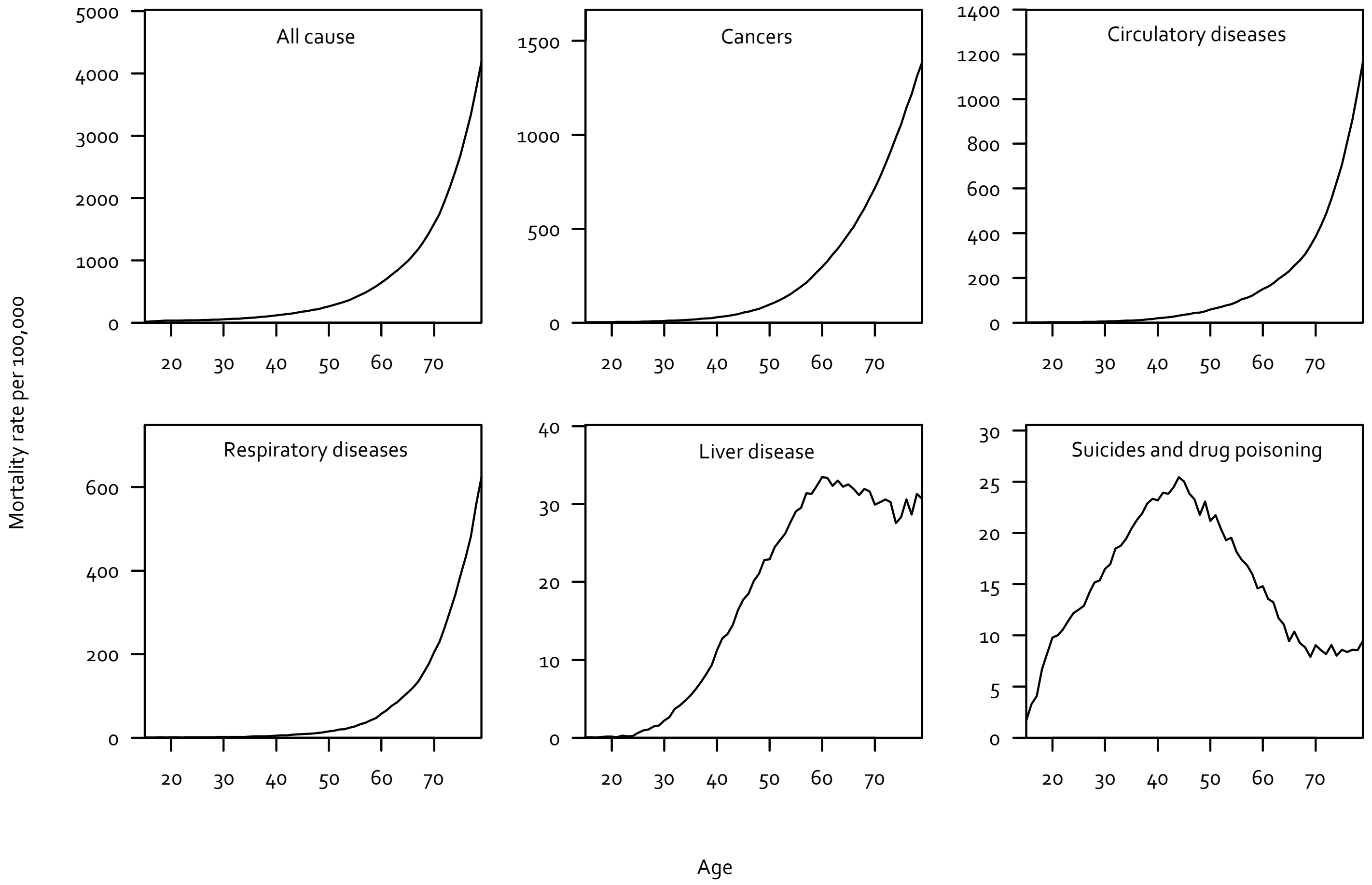

Numbers of deaths are from the Office for National Statistics[28] and population estimates are from Nomisweb. Data and code for this chart are available at https://github.com/danlewer/hupio/tree/main/general_pop_rates.

9. Association between age and cause-specific mortality in the matched comparison group

Figure 5: Association between age and cause-specific mortality rates

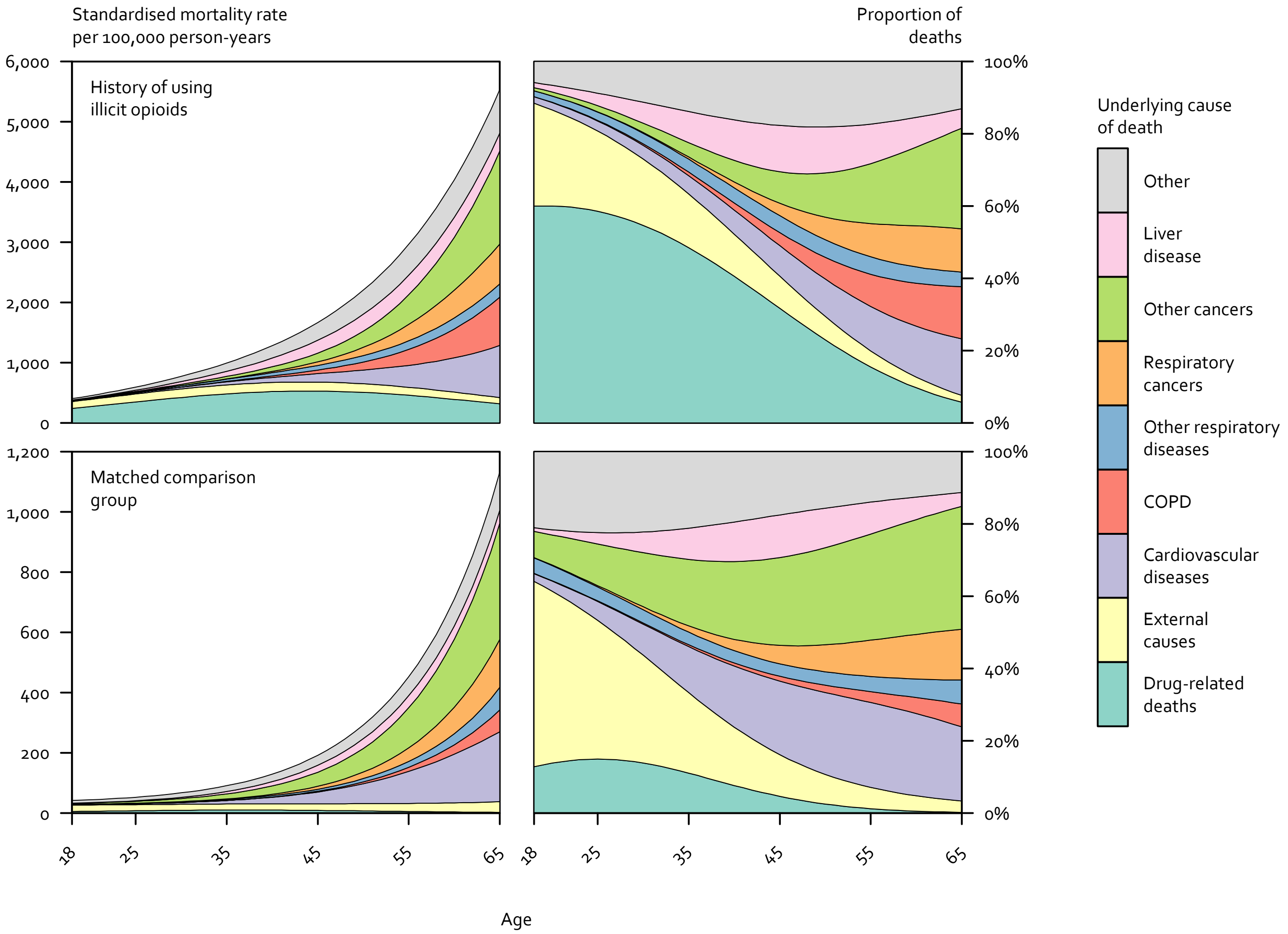

The top two panels in this figure replicate figure 3 in the main article, and the bottom two provide corresponding results for the matched comparison group (i.e. the general population).

10. Data used in charts in the article

Data and code for charts and tables in the article are provided at:
https://github.com/danlewer/hupio/tree/main/mortality

Table 7: Data from figure 2: mortality rates per 100,000 person-years (95% confidence interval) by time period, standardised for age, sex, and time after cohort entry

Period	All-cause	Drug poisoning	External	Circulatory	COPD	Other respiratory	Respiratory cancer	Other cancers	Liver	NCDs
History of using illicit opioids										
2001-03	1,335 (1,196-1,474)	409 (338-480)	151 (108-194)	125 (77-173)	43 (13-73)	42 (16-67)	88 (45-130)	142 (98-185)	88 (52-123)	527 (301-752)
2004-06	1,434 (1,309-1,558)	464 (396-533)	146 (110-182)	128 (90-167)	51 (26-75)	50 (28-73)	74 (42-106)	165 (121-209)	103 (72-135)	572 (379-765)
2007-09	1,435 (1,323-1,546)	426 (368-484)	138 (106-170)	134 (100-168)	62 (38-85)	71 (45-97)	63 (39-86)	147 (112-182)	150 (114-187)	627 (448-805)
2010-12	1,273 (1,179-1,367)	345 (299-391)	132 (103-161)	106 (80-132)	60 (40-80)	59 (38-80)	54 (36-73)	129 (100-158)	184 (143-224)	593 (438-748)
2013-15	1,397 (1,299-1,496)	490 (429-550)	146 (115-177)	126 (98-154)	76 (53-99)	51 (33-68)	57 (39-75)	117 (92-143)	128 (100-156)	556 (415-696)
2016-18	1,383 (1,287-1,479)	534 (468-600)	146 (115-177)	112 (88-137)	79 (56-103)	64 (43-86)	51 (35-68)	108 (85-131)	110 (85-134)	525 (391-658)
Comparison group										
2001-03	225 (189-261)	12 (4-20)	20 (12-29)	59 (37-81)	8 (0-17)	13 (3-22)	31 (11-51)	71 (47-95)	22 (10-34)	204 (108-301)
2004-06	210 (183-236)	11 (5-16)	27 (18-35)	42 (29-56)	5 (1-9)	9 (3-15)	22 (11-33)	54 (39-69)	12 (6-18)	144 (90-199)
2007-09	202 (180-224)	12 (7-17)	24 (17-31)	42 (31-53)	4 (1-7)	8 (4-13)	14 (8-20)	45 (35-56)	17 (11-23)	131 (89-172)
2010-12	183 (165-201)	9 (5-12)	23 (16-30)	36 (27-45)	4 (2-7)	5 (3-8)	12 (8-17)	48 (38-58)	15 (10-20)	120 (87-154)
2013-15	179 (163-196)	7 (4-9)	22 (16-28)	33 (25-40)	4 (2-7)	8 (4-11)	11 (7-15)	46 (37-55)	16 (11-21)	117 (86-148)
2016-18	162 (147-177)	7 (4-10)	18 (13-23)	26 (20-32)	6 (3-8)	7 (4-11)	9 (6-13)	41 (33-49)	13 (9-17)	103 (75-130)

COPD = chronic obstructive pulmonary disease
NCDs = non-communicable diseases, including circulatory, COPD, other respiratory, respiratory cancers, other cancers, and liver

Table 8: Data from figure 3 - mortality rates per 100,000 person-years (95% confidence interval) by age, standardised for time period, sex, and time after cohort entry

Age	All-cause	Drug poisoning	External	Circulatory	COPD	Other respiratory	Respiratory cancer	Other cancers	Liver	Other	NCDs
History of using illicit opioids											
18	410 (365-454)	241 (200-283)	115 (82-147)	7 (4-11)	0 (0-0)	6 (2-10)	0 (0-0)	4 (2-6)	6 (3-8)	23 (16-31)	23 (11-35)
19	430 (386-474)	256 (215-298)	117 (85-149)	8 (4-12)	0 (0-0)	7 (3-11)	0 (0-0)	4 (2-6)	7 (4-10)	26 (18-34)	27 (13-40)
20	452 (409-496)	271 (230-313)	120 (89-151)	9 (5-13)	0 (0-1)	8 (4-12)	0 (0-0)	5 (3-7)	8 (5-12)	30 (21-38)	31 (16-45)
21	476 (432-519)	286 (245-328)	123 (93-153)	10 (6-15)	0 (0-1)	9 (4-14)	0 (0-0)	6 (3-8)	10 (6-14)	33 (24-42)	36 (20-51)
22	500 (457-544)	302 (260-343)	125 (96-154)	12 (7-16)	1 (0-1)	10 (5-15)	0 (0-0)	7 (4-9)	12 (8-16)	37 (28-47)	41 (24-58)
23	526 (482-570)	317 (276-359)	128 (99-156)	13 (8-18)	1 (0-1)	11 (6-16)	0 (0-0)	8 (5-10)	15 (10-19)	42 (32-52)	47 (29-66)
24	553 (510-597)	333 (291-375)	130 (102-158)	15 (10-20)	1 (0-1)	12 (7-18)	0 (0-0)	9 (6-12)	17 (12-23)	47 (36-57)	54 (34-74)
25	582 (538-626)	348 (306-390)	132 (105-160)	17 (11-22)	1 (0-2)	14 (8-19)	0 (0-1)	10 (7-13)	21 (14-27)	52 (41-63)	62 (40-84)
26	613 (568-657)	364 (321-406)	134 (107-161)	19 (13-24)	1 (0-2)	15 (9-21)	0 (0-1)	12 (8-15)	24 (17-31)	58 (46-70)	71 (48-95)
27	645 (600-690)	379 (336-422)	136 (110-163)	21 (15-27)	2 (1-3)	17 (10-23)	1 (0-1)	13 (9-17)	28 (21-36)	64 (52-77)	82 (56-107)
28	679 (633-725)	394 (350-437)	138 (112-165)	23 (17-30)	2 (1-3)	19 (12-25)	1 (0-2)	15 (11-20)	33 (25-42)	71 (58-85)	93 (65-121)
29	715 (668-763)	408 (364-452)	140 (114-167)	26 (19-33)	3 (1-4)	20 (13-28)	1 (0-2)	18 (13-22)	39 (29-48)	79 (65-93)	107 (76-137)
30	753 (704-802)	422 (377-467)	142 (115-168)	29 (22-37)	3 (2-5)	22 (15-30)	2 (1-3)	20 (15-25)	45 (34-55)	87 (72-102)	121 (88-155)
31	794 (743-844)	436 (390-482)	143 (117-170)	33 (24-41)	4 (2-6)	25 (17-33)	2 (1-3)	23 (17-29)	51 (40-63)	96 (80-112)	138 (101-175)
32	836 (784-889)	448 (401-496)	145 (118-172)	36 (28-45)	5 (3-8)	27 (18-36)	3 (1-4)	26 (20-33)	59 (46-71)	105 (88-123)	157 (116-197)
33	881 (827-936)	461 (412-509)	146 (119-173)	41 (31-50)	6 (4-9)	30 (20-39)	4 (2-6)	30 (23-37)	67 (53-81)	115 (97-134)	177 (133-222)
34	929 (872-986)	472 (423-521)	147 (120-174)	45 (35-55)	8 (5-11)	32 (22-43)	5 (3-7)	35 (27-43)	76 (60-91)	126 (106-146)	201 (152-250)
35	980 (919-1,040)	483 (432-533)	148 (120-175)	50 (39-62)	10 (6-13)	35 (25-46)	6 (4-9)	40 (31-48)	85 (68-102)	138 (116-160)	227 (172-281)
36	1,033 (970-1,096)	492 (441-543)	149 (121-176)	56 (44-68)	12 (7-16)	39 (27-50)	8 (5-11)	45 (35-55)	96 (77-114)	150 (126-174)	255 (195-316)
37	1,090 (1,023-1,156)	501 (448-553)	149 (121-177)	62 (49-76)	14 (9-19)	42 (29-55)	10 (6-14)	52 (41-63)	107 (86-128)	163 (137-189)	287 (220-355)

Age	All-cause	Drug poisoning	External	Circulatory	COPD	Other respiratory	Respiratory cancer	Other cancers	Liver	Other	NCDs
38	1,150 (1,079-1,220)	508 (455-562)	150 (122-177)	69 (54-84)	17 (11-23)	46 (32-59)	13 (8-18)	59 (46-71)	118 (95-141)	177 (149-205)	323 (248-398)
39	1,213 (1,139-1,288)	515 (461-569)	150 (122-178)	77 (60-93)	21 (14-28)	50 (35-64)	17 (11-23)	67 (53-81)	131 (106-156)	191 (161-221)	362 (278-446)
40	1,281 (1,202-1,359)	520 (466-575)	150 (122-178)	85 (67-103)	25 (17-33)	54 (37-70)	21 (13-28)	76 (60-92)	144 (116-172)	207 (174-239)	405 (311-499)
41	1,352 (1,269-1,435)	524 (469-580)	150 (122-178)	94 (74-115)	30 (21-40)	58 (40-75)	26 (17-35)	87 (69-105)	157 (127-188)	223 (188-258)	453 (348-557)
42	1,428 (1,340-1,516)	527 (472-583)	150 (122-178)	104 (82-127)	36 (25-47)	62 (44-81)	32 (21-43)	99 (78-120)	171 (138-204)	240 (202-278)	505 (388-622)
43	1,508 (1,415-1,601)	529 (473-585)	149 (121-177)	115 (90-140)	43 (30-56)	67 (47-88)	39 (26-52)	112 (89-136)	185 (149-221)	258 (217-298)	563 (432-693)
44	1,593 (1,495-1,691)	530 (474-586)	149 (121-177)	128 (100-155)	51 (36-67)	72 (50-94)	48 (32-63)	128 (101-154)	200 (161-238)	276 (232-319)	626 (480-772)
45	1,683 (1,579-1,787)	529 (473-585)	148 (120-176)	141 (110-171)	61 (42-79)	78 (54-101)	58 (39-77)	145 (115-175)	214 (172-256)	295 (248-341)	696 (533-858)
46	1,779 (1,669-1,889)	527 (471-583)	147 (119-175)	155 (122-189)	71 (50-93)	83 (58-108)	70 (47-92)	164 (130-198)	228 (184-273)	315 (265-364)	772 (591-953)
47	1,880 (1,764-1,996)	524 (468-580)	146 (118-174)	171 (134-209)	84 (59-109)	89 (62-116)	83 (56-110)	186 (147-225)	242 (195-289)	335 (282-388)	855 (654-1,057)
48	1,988 (1,865-2,111)	520 (464-575)	145 (117-173)	189 (148-230)	98 (69-127)	95 (66-124)	99 (67-130)	211 (167-254)	256 (206-305)	356 (300-412)	947 (723-1,171)
49	2,102 (1,972-2,232)	514 (458-570)	143 (116-171)	208 (163-253)	114 (80-148)	101 (71-132)	116 (79-154)	238 (189-288)	269 (216-321)	377 (318-437)	1,047 (798-1,295)
50	2,223 (2,086-2,361)	507 (452-563)	142 (114-170)	229 (179-278)	132 (93-172)	108 (75-140)	136 (93-180)	269 (214-325)	281 (226-335)	399 (336-462)	1,155 (880-1,431)
51	2,352 (2,207-2,498)	500 (444-555)	140 (112-168)	251 (197-306)	153 (107-199)	114 (80-149)	159 (108-210)	304 (242-367)	292 (235-349)	421 (355-488)	1,274 (969-1,579)
52	2,489 (2,335-2,643)	491 (435-546)	138 (110-167)	276 (217-335)	176 (124-229)	121 (85-158)	184 (125-242)	343 (273-414)	302 (243-361)	444 (374-514)	1,403 (1,066-1,739)
53	2,634 (2,470-2,798)	481 (426-537)	137 (108-165)	303 (238-368)	203 (142-263)	128 (90-167)	211 (144-278)	387 (308-466)	311 (250-372)	467 (393-541)	1,543 (1,172-1,914)
54	2,789 (2,614-2,964)	471 (415-526)	134 (106-163)	332 (261-404)	232 (163-300)	136 (94-177)	241 (165-317)	436 (347-525)	319 (255-382)	490 (412-568)	1,695 (1,285-2,105)
55	2,953 (2,766-3,140)	459 (403-515)	132 (103-162)	364 (286-442)	264 (186-342)	143 (99-187)	273 (187-359)	491 (391-591)	325 (259-390)	513 (430-595)	1,860 (1,408-2,311)
56	3,127 (2,927-3,327)	447 (390-503)	130 (100-160)	398 (312-485)	300 (212-388)	151 (104-197)	307 (210-403)	553 (440-665)	329 (262-397)	536 (448-623)	2,038 (1,541-2,536)
57	3,313 (3,097-3,528)	434 (377-491)	128 (97-158)	436 (341-531)	339 (240-439)	158 (109-208)	343 (235-451)	621 (495-748)	332 (263-402)	559 (466-651)	2,231 (1,682-2,779)
58	3,510 (3,277-3,743)	420 (363-478)	125 (94-156)	476 (371-581)	382 (270-495)	166 (113-220)	381 (261-501)	698 (555-842)	334 (262-405)	581 (483-679)	2,438 (1,832-3,044)
59	3,719 (3,466-3,972)	406 (348-465)	123 (91-155)	520 (404-636)	430 (302-557)	174 (117-231)	421 (287-554)	784 (621-946)	333 (259-407)	603 (498-708)	2,662 (1,991-3,332)
60	3,942 (3,666-4,218)	392 (332-451)	120 (87-153)	568 (438-697)	481 (337-624)	182 (121-244)	461 (313-609)	879 (694-1,064)	331 (255-408)	625 (513-737)	2,902 (2,158-3,646)
61	4,179 (3,877-4,481)	377 (317-437)	117 (83-151)	619 (474-764)	536 (373-699)	190 (124-257)	502 (338-666)	985 (774-1,197)	328 (249-407)	646 (526-766)	3,160 (2,332-3,988)
62	4,432 (4,099-4,764)	362 (301-423)	115 (80-150)	674 (512-837)	595 (410-780)	198 (127-270)	543 (361-725)	1,104 (861-1,346)	322 (241-404)	667 (538-795)	3,437 (2,511-4,363)
63	4,700 (4,333-5,067)	346 (285-408)	112 (76-148)	734 (551-917)	659 (447-870)	207 (129-284)	583 (381-786)	1,235 (955-1,515)	316 (231-400)	686 (548-825)	3,733 (2,694-4,772)
64	4,986 (4,579-5,393)	331 (268-393)	109 (72-146)	798 (591-1,006)	726 (484-968)	215 (130-299)	623 (397-848)	1,381 (1,057-1,706)	307 (221-394)	705 (556-854)	4,050 (2,880-5,221)
65	5,290 (4,838-5,743)	315 (252-378)	106 (68-144)	868 (632-1,103)	798 (520-1,075)	223 (130-315)	660 (409-912)	1,544 (1,166-1,921)	298 (209-387)	723 (562-884)	4,389 (3,066-5,713)
Comparison group											
18	36 (30-43)	5 (2-9)	21 (13-30)	1 (0-1)	0 (0-0)	2 (0-3)	0 (0-0)	3 (2-4)	0 (0-1)	9 (5-12)	6 (2-10)
19	38 (31-45)	6 (2-9)	21 (13-29)	1 (0-2)	0 (0-0)	2 (0-3)	0 (0-0)	3 (2-5)	1 (0-1)	9 (5-13)	7 (3-11)
20	40 (33-46)	6 (2-10)	21 (14-28)	1 (1-2)	0 (0-0)	2 (0-3)	0 (0-0)	4 (2-5)	1 (0-1)	10 (6-13)	8 (3-12)
21	42 (35-48)	6 (3-10)	21 (14-28)	1 (1-2)	0 (0-0)	2 (0-3)	0 (0-0)	4 (3-6)	1 (0-1)	10 (6-13)	8 (4-13)
22	44 (37-50)	7 (3-11)	21 (14-27)	2 (1-3)	0 (0-0)	2 (1-3)	0 (0-0)	5 (3-6)	1 (0-2)	10 (7-14)	9 (5-14)
23	46 (40-53)	7 (4-11)	20 (14-27)	2 (1-3)	0 (0-0)	2 (1-3)	0 (0-0)	5 (3-7)	1 (0-2)	11 (7-14)	10 (5-15)
24	48 (42-55)	8 (4-11)	20 (14-26)	2 (1-3)	0 (0-0)	2 (1-3)	0 (0-0)	6 (4-7)	1 (1-2)	11 (8-15)	11 (6-17)
25	51 (45-58)	8 (4-12)	20 (14-26)	3 (2-4)	0 (0-0)	2 (1-3)	0 (0-0)	6 (4-8)	2 (1-3)	12 (8-15)	13 (7-18)
26	54 (47-61)	8 (4-12)	20 (14-26)	3 (2-4)	0 (0-0)	2 (1-3)	0 (0-0)	7 (5-9)	2 (1-3)	12 (9-16)	14 (9-20)
27	57 (50-64)	9 (5-12)	20 (15-26)	4 (2-5)	0 (0-0)	2 (1-3)	0 (0-1)	7 (5-9)	2 (1-3)	13 (9-16)	16 (10-22)
28	60 (53-67)	9 (5-13)	20 (15-26)	4 (3-6)	0 (0-0)	2 (1-4)	0 (0-1)	8 (6-10)	3 (2-4)	14 (10-17)	18 (11-25)
29	64 (57-71)	9 (5-13)	20 (15-25)	5 (3-7)	0 (0-0)	2 (1-4)	0 (0-1)	9 (7-11)	3 (2-5)	14 (11-18)	20 (13-27)
30	68 (61-75)	9 (5-13)	20 (15-25)	6 (4-8)	0 (0-0)	2 (1-4)	1 (0-1)	10 (8-12)	4 (2-5)	15 (11-19)	23 (15-31)
31	72 (64-80)	10 (6-13)	20 (15-25)	7 (5-9)	0 (0-1)	3 (1-4)	1 (0-1)	11 (9-14)	4 (3-6)	16 (12-19)	26 (17-34)
32	77 (69-84)	10 (6-14)	20 (15-25)	8 (6-10)	0 (0-1)	3 (1-4)	1 (0-1)	12 (10-15)	5 (3-7)	16 (13-20)	29 (20-38)
33	82 (73-90)	10 (6-14)	20 (15-25)	9 (7-11)	0 (0-1)	3 (1-4)	1 (0-2)	14 (11-17)	6 (4-8)	17 (13-21)	33 (23-43)
34	87 (78-96)	10 (6-14)	20 (15-25)	10 (8-13)	0 (0-1)	3 (1-4)	1 (1-2)	15 (12-18)	7 (4-9)	18 (14-23)	37 (26-48)
35	93 (84-102)	10 (6-14)	20 (15-26)	12 (9-14)	0 (0-1)	3 (2-5)	2 (1-2)	17 (13-20)	8 (5-11)	19 (15-24)	41 (29-53)
36	99 (89-109)	10 (6-14)	20 (15-26)	13 (10-16)	1 (0-1)	3 (2-5)	2 (1-3)	18 (15-22)	9 (6-12)	20 (16-25)	46 (33-60)
37	106 (96-116)	10 (6-14)	20 (15-26)	15 (11-19)	1 (0-1)	4 (2-5)	2 (1-3)	20 (16-25)	10 (7-13)	21 (16-26)	52 (38-67)
38	114 (103-125)	10 (6-14)	21 (15-26)	17 (13-21)	1 (0-1)	4 (2-6)	3 (2-4)	23 (18-27)	11 (8-15)	23 (17-28)	59 (42-75)
39	122 (110-134)	10 (6-14)	21 (15-26)	19 (15-24)	1 (0-2)	4 (2-6)	3 (2-5)	25 (20-30)	13 (9-17)	24 (18-29)	66 (48-84)
40	131 (118-144)	10 (6-14)	21 (15-26)	22 (17-27)	1 (0-2)	4 (2-7)	4 (2-6)	28 (22-33)	14 (10-19)	25 (19-31)	74 (53-94)
41	141 (127-155)	10 (6-14)	21 (16-27)	25 (19-30)	1 (0-2)	5 (2-7)	5 (3-7)	31 (25-37)	16 (11-21)	27 (20-33)	82 (60-105)
42	152 (137-167)	10 (6-14)	21 (16-27)	28 (21-34)	2 (0-3)	5 (3-8)	6 (3-8)	34 (27-41)	17 (12-23)	28 (22-35)	92 (67-117)

Age	All-cause	Drug poisoning	External	Circulatory	COPD	Other respiratory	Respiratory cancer	Other cancers	Liver	Other	NCDs
43	164 (148-180)	9 (5-13)	22 (16-27)	31 (24-38)	2 (1-3)	6 (3-8)	7 (4-10)	38 (30-46)	19 (13-25)	30 (23-37)	103 (75-131)
44	177 (159-194)	9 (5-13)	22 (16-28)	35 (27-43)	2 (1-4)	6 (3-9)	8 (5-12)	42 (34-51)	21 (14-28)	32 (24-39)	114 (83-146)
45	191 (172-210)	9 (5-13)	22 (16-28)	39 (30-48)	3 (1-4)	7 (3-10)	10 (6-14)	47 (37-56)	23 (15-30)	34 (26-41)	128 (93-162)
46	207 (186-227)	9 (5-12)	22 (16-29)	44 (34-54)	3 (1-5)	7 (4-11)	12 (7-16)	52 (41-62)	25 (17-33)	36 (27-44)	142 (103-181)
47	224 (202-246)	8 (5-12)	23 (17-29)	49 (37-60)	4 (1-6)	8 (4-12)	14 (8-19)	57 (46-69)	26 (18-35)	38 (29-47)	158 (114-201)
48	243 (219-267)	8 (5-11)	23 (17-29)	54 (42-67)	4 (1-7)	9 (4-13)	16 (10-22)	64 (51-77)	28 (19-38)	40 (31-50)	175 (127-223)
49	264 (238-290)	8 (4-11)	24 (17-30)	60 (46-74)	5 (2-9)	10 (5-15)	19 (11-26)	71 (57-85)	30 (20-40)	43 (33-53)	194 (141-248)
50	287 (259-315)	7 (4-11)	24 (17-31)	66 (51-82)	6 (2-10)	11 (6-16)	22 (13-31)	79 (63-94)	32 (22-43)	46 (35-56)	216 (156-275)
51	313 (282-343)	7 (4-10)	24 (18-31)	73 (56-90)	7 (2-12)	12 (6-18)	25 (15-36)	87 (70-105)	34 (23-45)	49 (37-60)	239 (173-305)
52	341 (308-374)	7 (3-10)	25 (18-32)	81 (62-99)	8 (3-14)	13 (7-20)	29 (18-41)	97 (78-116)	36 (24-47)	52 (40-64)	264 (191-338)
53	372 (336-408)	6 (3-9)	25 (18-33)	89 (68-109)	10 (3-17)	15 (8-22)	34 (21-48)	108 (86-129)	37 (25-49)	55 (42-68)	293 (211-374)
54	407 (367-446)	6 (3-9)	26 (19-33)	97 (75-120)	12 (4-19)	17 (9-25)	39 (24-55)	120 (96-143)	39 (26-51)	59 (45-73)	324 (233-414)
55	445 (402-488)	5 (3-8)	27 (19-34)	106 (82-131)	14 (4-23)	19 (10-28)	45 (27-63)	133 (107-159)	40 (27-53)	63 (48-78)	358 (257-458)
56	488 (440-536)	5 (2-8)	27 (19-35)	116 (89-143)	16 (5-27)	22 (11-32)	52 (32-73)	148 (119-177)	41 (27-55)	67 (51-84)	395 (283-507)
57	535 (483-588)	5 (2-8)	28 (19-37)	127 (97-156)	19 (6-32)	25 (13-36)	60 (36-83)	164 (132-197)	42 (28-57)	72 (55-90)	436 (312-561)
58	588 (530-647)	4 (2-7)	29 (20-38)	138 (106-170)	22 (7-38)	28 (14-42)	68 (41-95)	183 (146-219)	43 (28-58)	77 (58-96)	482 (343-621)
59	647 (582-712)	4 (1-7)	29 (20-39)	149 (114-185)	27 (9-44)	32 (16-48)	78 (47-108)	203 (162-244)	44 (28-59)	83 (62-104)	532 (376-688)
60	712 (639-786)	4 (1-6)	30 (20-41)	162 (123-201)	31 (10-53)	36 (18-55)	88 (53-123)	226 (180-272)	44 (28-61)	89 (66-112)	588 (412-764)
61	786 (703-868)	3 (1-6)	31 (20-42)	175 (132-218)	37 (12-62)	42 (21-63)	100 (59-140)	251 (199-304)	45 (28-61)	95 (70-121)	649 (450-848)
62	867 (774-961)	3 (1-6)	32 (20-44)	188 (141-236)	44 (13-74)	48 (23-73)	113 (66-159)	280 (220-339)	45 (27-62)	102 (74-130)	717 (490-944)
63	959 (852-1,066)	3 (0-5)	33 (20-46)	203 (149-256)	52 (15-88)	56 (26-85)	127 (73-180)	311 (243-380)	44 (26-63)	110 (79-141)	792 (532-1,052)
64	1,062 (938-1,185)	3 (0-5)	34 (20-48)	217 (158-277)	61 (16-106)	65 (29-100)	142 (80-205)	347 (268-425)	44 (25-63)	118 (83-153)	876 (576-1,175)
65	1,177 (1,034-1,319)	2 (0-5)	35 (20-50)	233 (166-299)	72 (17-127)	75 (32-118)	159 (87-232)	386 (295-477)	43 (23-63)	127 (88-167)	968 (621-1,316)

COPD = chronic obstructive pulmonary disease
NCDs = non-communicable diseases, including circulatory, COPD, other respiratory, respiratory cancers, other cancers, and liver

Table 9: Data from figure 4: marginal mortality rates for age in the present study, applied to the age structure of participants in the Unlinked Anonymous Monitoring Survey of People who Inject Drugs. Deaths per 100,000 person-years (95% confidence interval)

Calendar year	All-cause	Drug poisoning	External	Circulatory	COPD	Other respiratory	Respiratory cancer	Other cancers	Liver	Other	NCDs
2001	751 (691-811)	396 (343-449)	138 (109-167)	36 (26-46)	12 (6-18)	24 (14-34)	10 (6-13)	39 (28-49)	50 (38-63)	105 (83-127)	171 (119-223)
2002	810 (749-871)	408 (356-460)	138 (110-167)	42 (31-53)	14 (8-20)	27 (17-38)	12 (8-17)	45 (34-56)	62 (47-76)	116 (94-139)	203 (145-260)
2003	856 (792-919)	411 (359-463)	139 (110-167)	48 (36-61)	19 (11-26)	30 (19-41)	17 (11-22)	53 (40-65)	67 (51-82)	123 (100-147)	233 (169-297)
2004	824 (762-886)	410 (358-462)	138 (110-167)	44 (33-55)	15 (9-22)	28 (18-39)	13 (9-18)	47 (36-59)	63 (48-78)	119 (96-141)	211 (152-270)
2005	850 (787-913)	413 (362-465)	138 (110-166)	47 (35-59)	17 (10-24)	30 (19-41)	15 (10-21)	51 (39-63)	67 (51-82)	123 (100-146)	227 (165-289)
2006	849 (786-911)	414 (363-466)	138 (110-166)	47 (35-59)	17 (10-23)	30 (19-40)	15 (10-20)	50 (38-62)	67 (52-82)	123 (100-146)	225 (164-286)
2007	835 (773-897)	413 (361-464)	138 (110-166)	45 (34-57)	16 (9-22)	29 (18-39)	14 (9-19)	49 (37-60)	65 (50-79)	121 (98-143)	217 (157-277)
2008	868 (806-931)	421 (369-472)	138 (111-166)	49 (37-61)	17 (10-24)	31 (20-42)	15 (10-20)	51 (39-63)	71 (55-87)	127 (104-151)	234 (171-296)
2009	929 (863-995)	425 (373-476)	139 (111-167)	57 (43-70)	22 (14-31)	34 (22-45)	20 (14-27)	61 (47-75)	79 (62-96)	137 (112-162)	273 (202-344)
2010	946 (881-1,012)	432 (380-483)	140 (112-167)	58 (45-72)	22 (14-30)	35 (23-47)	20 (13-26)	61 (48-75)	83 (66-101)	141 (116-166)	279 (208-351)
2011	992 (925-1,060)	437 (386-488)	140 (113-167)	64 (49-78)	26 (17-35)	37 (25-50)	24 (16-31)	68 (53-83)	89 (71-108)	149 (123-174)	308 (231-385)
2012	981 (915-1,048)	438 (387-490)	140 (113-167)	62 (48-76)	24 (15-32)	37 (25-49)	22 (15-29)	65 (51-79)	89 (71-108)	148 (122-173)	299 (224-374)
2013	1,034 (965-1,102)	442 (391-493)	140 (113-167)	69 (54-84)	29 (19-38)	40 (27-53)	27 (18-35)	74 (58-89)	95 (76-115)	156 (129-182)	333 (251-415)
2014	1,062 (992-1,132)	449 (398-501)	141 (114-168)	72 (56-88)	28 (19-38)	41 (28-54)	27 (18-35)	76 (60-91)	103 (82-124)	163 (136-190)	346 (262-430)
2015	1,157 (1,082-1,232)	455 (404-507)	141 (115-168)	85 (66-103)	37 (26-49)	46 (31-60)	35 (24-47)	92 (73-110)	114 (91-136)	178 (149-207)	408 (311-506)
2016	1,201 (1,125-1,278)	463 (411-514)	142 (115-168)	90 (71-109)	40 (27-52)	48 (33-63)	38 (26-49)	97 (77-117)	122 (98-146)	187 (157-217)	433 (332-535)
2017	1,251 (1,171-1,330)	464 (412-516)	142 (116-169)	97 (76-117)	45 (31-59)	51 (35-66)	43 (30-56)	105 (84-126)	126 (101-151)	194 (163-225)	467 (357-577)
2018	1,284 (1,203-1,364)	468 (416-521)	143 (116-169)	101 (80-122)	47 (32-61)	52 (36-68)	45 (31-59)	110 (88-132)	132 (106-158)	201 (169-232)	487 (373-600)

COPD = chronic obstructive pulmonary disease
NCDs = non-communicable diseases, including circulatory, COPD, other respiratory, respiratory cancers, other cancers, and liver

11. STROBE checklist

	Item No	Recommendation	Page No
Title and abstract	1	(*a*) Indicate the study's design with a commonly used term in the title or the abstract	Title – p1
		(*b*) Provide in the abstract an informative and balanced summary of what was done and what was found	Abstract – p2
Introduction			
Background/rationale	2	Explain the scientific background and rationale for the investigation being reported	Introduction – p5
Objectives	3	State specific objectives, including any prespecified hypotheses	Introduction – p5
Methods			
Study design	4	Present key elements of study design early in the paper	Methods/study design – p6
Setting	5	Describe the setting, locations, and relevant dates, including periods of recruitment, exposure, follow-up, and data collection	Methods/data source – pp6-7
Participants	6	(*a*) *Cohort study*—Give the eligibility criteria, and the sources and methods of selection of participants. Describe methods of follow-up<br>*Case-control study*—Give the eligibility criteria, and the sources and methods of case ascertainment and control selection. Give the rationale for the choice of cases and controls<br>*Cross-sectional study*—Give the eligibility criteria, and the sources and methods of selection of participants	Methods/data source – pp6-7
		(*b*) *Cohort study*—For matched studies, give matching criteria and number of exposed and unexposed<br>*Case-control study*—For matched studies, give matching criteria and the number of controls per case	Methods/data source – p6
Variables	7	Clearly define all outcomes, exposures, predictors, potential confounders, and effect modifiers. Give diagnostic criteria, if applicable	Methods/analysis – pp7-8
Data sources/ measurement	8*	For each variable of interest, give sources of data and details of methods of assessment (measurement). Describe comparability of assessment methods if there is more than one group	Methods/data source – pp6-7<br>Methods/cause of death grouping – p7
Bias	9	Describe any efforts to address potential sources of bias	Methods/data source (exposure density sampling) – p6
Study size	10	Explain how the study size was arrived at	Supplementary Information – p7 (flow chart)
Quantitative variables	11	Explain how quantitative variables were handled in the analyses. If applicable, describe which groupings were chosen and why	Methods/cause of death grouping – p7
Statistical methods	12	(*a*) Describe all statistical methods, including those used to control for confounding	Methods/analysis – pp7-8

	Item No	Recommendation	Page No
		(*b*) Describe any methods used to examine subgroups and interactions	Methods/analysis – pp7-8 (interactions between opioid use and calendar time period / duration in cohort)
		(*c*) Explain how missing data were addressed	NA (missing data only in variables used for descriptive purposes)
		(*d*) *Cohort study*—If applicable, explain how loss to follow-up was addressed<br>*Case-control study*—If applicable, explain how matching of cases and controls was addressed<br>*Cross-sectional study*—If applicable, describe analytical methods taking account of sampling strategy	NA (complete data linkage for participants in the study)
		(*e*) Describe any sensitivity analyses	NA
Results			
Participants	13*	(a) Report numbers of individuals at each stage of study—eg numbers potentially eligible, examined for eligibility, confirmed eligible, included in the study, completing follow-up, and analysed	Supplementary Information – p7 (flow chart)
		(b) Give reasons for non-participation at each stage	Supplementary Information – p7 (flow chart)
		(c) Consider use of a flow diagram	Supplementary Information – p7 (flow chart)
Descriptive data	14*	(a) Give characteristics of study participants (eg demographic, clinical, social) and information on exposures and potential confounders	Results/table 1 – p10
		(b) Indicate number of participants with missing data for each variable of interest	NA (missing data only in variables used for descriptive purposes)<br>Missing data for descriptive variables shown in Results/table 1 – p10
		(c) *Cohort study*—Summarise follow-up time (eg, average and total amount)	Results/table 1 – p10
Outcome data	15*	*Cohort study*—Report numbers of outcome events or summary measures over time	Results – p9<br>Results/table 1 – p10
		Case-control study—Report numbers in each exposure category, or summary measures of exposure	-
		Cross-sectional study—Report numbers of outcome events or summary measures	-
Main results	16	(*a*) Give unadjusted estimates and, if applicable, confounder-adjusted estimates and their precision (eg, 95% confidence interval). Make clear which confounders were adjusted for and why they were included	Results – p11-12<br>(Note that unadjusted estimates are not available because participants are matched on age, sex, and cohort entry date, i.e. confounding is handled in the design rather than analysis)
		(*b*) Report category boundaries when continuous variables were categorized	NA
		(*c*) If relevant, consider translating estimates of relative risk into absolute risk for a meaningful time period	Results /Figures 2-4 (results are presented in terms of standardised rates)

	Item No	Recommendation	Page No
Other analyses	17	Report other analyses done—eg analyses of subgroups and interactions, and sensitivity analyses	NA
Discussion			
Key results	18	Summarise key results with reference to study objectives	Discussion – p13
Limitations	19	Discuss limitations of the study, taking into account sources of potential bias or imprecision. Discuss both direction and magnitude of any potential bias	Discussion – p14
Interpretation	20	Give a cautious overall interpretation of results considering objectives, limitations, multiplicity of analyses, results from similar studies, and other relevant evidence	Discussion – p13
Generalisability	21	Discuss the generalisability (external validity) of the study results	Discussion – p14
Other information			
Funding	22	Give the source of funding and the role of the funders for the present study and, if applicable, for the original study on which the present article is based	Methods – p8<br>Additional information – p15

12. References for supplementary information

1. Larney S, Tran LT, Leung J, Santo T, Santomauro D, Hickman M, et al. All-Cause and Cause-Specific Mortality Among People Using Extramedical Opioids: A Systematic Review and Meta-analysis. JAMA Psychiatry. 2020;77: 493. doi:10.1001/jamapsychiatry.2019.4170

2. Brugal MT, Domingo-Salvany A, Puig R, Barrio G, García de Olalla P, de la Fuente L. Evaluating the impact of methadone maintenance programmes on mortality due to overdose and aids in a cohort of heroin users in Spain. Addiction. 2005;100: 981–989. doi:10.1111/j.1360-0443.2005.01089.x

3. McDonald S, McAuley A, Hickman M, Bird S, Weir A, Templeton K, et al. Increasing drug-related mortality rates over the last decade in Scotland are not just due to an ageing cohort: A retrospective longitudinal cohort study. Int J Drug Policy. 2021; 103286. doi:10.1016/j.drugpo.2021.103286

4. Pearce LA, Min JE, Piske M, Zhou H, Homayra F, Slaunwhite A, et al. Opioid agonist treatment and risk of mortality during opioid overdose public health emergency: population based retrospective cohort study. BMJ. 2020; m772. doi:10.1136/bmj.m772

5. Gao L, Robertson JR, Bird SM. Non drug-related and opioid-specific causes of 3262 deaths in Scotland's methadone-prescription clients, 2009–2015. Drug Alcohol Depend. 2019;197: 262–270. doi:10.1016/j.drugalcdep.2019.01.019

6. Molist G, Brugal MT, Barrio G, Mesías B, Bosque-Prous M, Parés-Badell O, et al. Effect of ageing and time since first heroin and cocaine use on mortality from external and natural causes in a Spanish cohort of drug users. Int J Drug Policy. 2018;53: 8–16. doi:10.1016/j.drugpo.2017.11.011

7. Pavarin RM, Fioritti A, Sanchini S. Mortality trends among heroin users treated between 1975 and 2013 in Northern Italy: Results of a longitudinal study. J Subst Abuse Treat. 2017;77: 166–173. doi:10.1016/j.jsat.2017.02.009

8. Jerkeman A, Håkansson A, Rylance R, Wagner P, Alanko Blomé M, Björkman P. Death from liver disease in a cohort of injecting opioid users in a Swedish city in relation to registration for opioid substitution therapy: Liver mortality in opiate substitution therapy. Drug Alcohol Rev. 2017;36: 424–431. doi:10.1111/dar.12425

9. Nambiar D, Weir A, Aspinall EJ, Stoové M, Hutchinson S, Dietze P, et al. Mortality and cause of death in a cohort of people who had ever injected drugs in Glasgow: 1982–2012. Drug Alcohol Depend. 2015;147: 215–221. doi:10.1016/j.drugalcdep.2014.11.008

10. Larney S, Bohnert ASB, Ganoczy D, Ilgen MA, Hickman M, Blow FC, et al. Mortality among older adults with opioid use disorders in the Veteran's Health Administration, 2000–2011. Drug Alcohol Depend. 2015;147: 32–37. doi:10.1016/j.drugalcdep.2014.12.019

11. Pierce M, Bird SM, Hickman M, Millar T. National record linkage study of mortality for a large cohort of opioid users ascertained by drug treatment or criminal justice sources in England, 2005–2009. Drug Alcohol Depend. 2015;146: 17–23. doi:10.1016/j.drugalcdep.2014.09.782

12. Vajdic CM, Pour SM, Olivier J, Swart A, O'Connell DL, Falster MO, et al. The impact of blood-borne viruses on cause-specific mortality among opioid dependent people: An Australian population-based cohort study. Drug Alcohol Depend. 2015;152: 264–271. doi:10.1016/j.drugalcdep.2015.03.026

13. Onyeka IN, Beynon CM, Hannila M-L, Tiihonen J, Föhr J, Tuomola P, et al. Patterns and 14-year trends in mortality among illicit drug users in Finland: The HUUTI study. Int J Drug Policy. 2014;25: 1047–1053. doi:10.1016/j.drugpo.2014.07.008

14. Degenhardt L, Larney S, Randall D, Burns L, Hall W. Causes of death in a cohort treated for opioid dependence between 1985 and 2005: Mortality among opioid-dependent people. Addiction. 2014;109: 90–99. doi:10.1111/add.12337

15. Kielland KB, Skaug K, Amundsen EJ, Dalgard O. All-cause and liver-related mortality in hepatitis C infected drug users followed for 33 years: A controlled study. J Hepatol. 2013;58: 31–37. doi:10.1016/j.jhep.2012.08.024

16. Gibson A, Randall D, Degenhardt L. The increasing mortality burden of liver disease among opioid-dependent people: cohort study: Liver mortality among opioid-dependent people. Addiction. 2011;106: 2186–2192. doi:10.1111/j.1360-0443.2011.03575.x

17. Stenbacka M, Leifman A, Romelsjö A. Mortality and cause of death among 1705 illicit drug users: A 37 year follow up: Mortality among illicit drug users. Drug Alcohol Rev. 2009;29: 21–27. doi:10.1111/j.1465-3362.2009.00075.x

18. Beynon C, McVeigh J, Hurst A, Marr A. Older and sicker: Changing mortality of drug users in treatment in the North West of England. Int J Drug Policy. 2010;21: 429–431. doi:10.1016/j.drugpo.2010.01.012

19. Ferreros I, Lumbreras B, Hurtado I, Pérez-Hoyos S, Hernández-Aguado I. The shifting pattern of cause-specific mortality in a cohort of human immunodeficiency virus-infected and non-infected injecting drug users. Addiction. 2008;103: 651–659. doi:10.1111/j.1360-0443.2008.02135.x

20. Maxwell JC, Pullum TW, Tannert K. Deaths of clients in methadone treatment in Texas: 1994–2002. Drug Alcohol Depend. 2005;78: 73–81. doi:10.1016/j.drugalcdep.2004.09.006

21. Copeland L, Budd J, Robertson JR, Elton RA. Changing Patterns in Causes of Death in a Cohort of Injecting Drug Users, 1980-2001. Arch Intern Med. 2004;164: 1214. doi:10.1001/archinte.164.11.1214

22. Bargagli AM, Sperati A, Davoli M, Forastiere F, Perucci CA. Mortality among problem drug users in Rome: an 18-year follow-up study, 1980-97. Addiction. 2001;96: 1455–1463. doi:10.1046/j.1360-0443.2001.961014559.x

23. Office for National Statistics. Deaths related to drug poisoning in England and Wales: 2019 registrations. 2020. Available: https://www.ons.gov.uk/peoplepopulationandcommunity/birthsdeathsandmarriages/deaths/bulletins/deathsrelatedtodrugpoisoningienglandandwales/2019registrations

24. Lewer D, Jayatunga W, Aldridge RW, Edge C, Marmot M, Story A, et al. Premature mortality attributable to socioeconomic inequality in England between 2003 and 2018: an observational study. Lancet Public Health. 2020;5: e33–e41. doi:10.1016/S2468-2667(19)30219-1

25. Lewer D, Tweed EJ, Aldridge RW, Morley KI. Causes of hospital admission and mortality among 6683 people who use heroin and crack cocaine, comparing relative and absolute risks: a population-based cohort study. Drug Alcohol Depend. 2019;204. doi:10.1016/j.drugalcdep.2019.06.027

26. Public Health England. People who inject drugs: HIV and viral hepatitis monitoring. 2021. Available: https://www.gov.uk/government/publications/people-who-inject-drugs-hiv-and-viral-hepatitis-monitoring

27. Public Health England. Substance misuse treatment for adults: statistics 2019 to 2020. 2020. Available: https://www.gov.uk/government/statistics/substance-misuse-treatment-for-adults-statistics-2019-to-2020

28. Office for National Statistics. Deaths by age, sex, and selected underlying causes, people aged 15 to 79 years, England & Wales and usual residents of London, deaths registered in 2001 to 2019. 2021. Available: https://www.ons.gov.uk/peoplepopulationandcommunity/birthsdeathsandmarriages/deaths/adhocs/13123deathsbyagesexandselectedunderlyingcausespeopleaged15to79yearsenglandwalesandusualresidentsoflondondeathsregisteredin2001to2019
